# Bioinformatic analysis of *Entamoeba histolytica *SINE1 elements

**DOI:** 10.1186/1471-2164-11-321

**Published:** 2010-05-24

**Authors:** Derek M Huntley, Ioannis Pandis, Sarah A Butcher, John P Ackers

**Affiliations:** 1 Centre for Bioinformatics, Division of Molecular Biosciences, Imperial College London, London, SW7 2AZ , UK; 2 Department of Infectious & Tropical Diseases, London School of Hygiene & Tropical Medicine, Keppel Street, London, WC1E 7HT, UK; 3 Current Address: Institute of Immunology, Biomedical Sciences Research Center (BSRC) "Alexander Fleming", Vari 16672, Greece

## Abstract

**Background:**

Invasive amoebiasis, caused by infection with the human parasite *Entamoeba histolytica *remains a major cause of morbidity and mortality in some less-developed countries. Genetically *E. histolytica *exhibits a number of unusual features including having approximately 20% of its genome comprised of repetitive elements. These include a number of families of SINEs - non-autonomous elements which can, however, move with the help of partner LINEs. In many eukaryotes SINE mobility has had a profound effect on gene expression; in this study we concentrated on one such element - EhSINE1, looking in particular for evidence of recent transposition.

**Results:**

EhSINE1s were detected in the newly reassembled *E. histolytica *genome by searching with a Hidden Markov Model developed to encapsulate the key features of this element; 393 were detected. Examination of their sequences revealed that some had an internal structure showing one to four 26-27 nt repeats. Members of the different classes differ in a number of ways and in particular those with two internal repeats show the properties expected of fairly recently transposed SINEs - they are the most homogeneous in length and sequence, they have the longest (i.e. the least decayed) target site duplications and are the most likely to show evidence (in a cDNA library) of active transcription. Furthermore we were able to identify 15 EhSINE1s (6 pairs and one triplet) which appeared to be identical or very nearly so but inserted into different sites in the genome; these provide good evidence that if mobility has now ceased it has only done so very recently.

**Conclusions:**

Of the many families of repetitive elements present in the genome of *E. histolytica *we have examined in detail just one - EhSINE1. We have shown that there is evidence for waves of transposition at different points in the past and no evidence that mobility has entirely ceased. There are many aspects of the biology of this parasite which are not understood, in particular why it is pathogenic while the closely related species *E. dispar *is not, the great genetic diversity found amongst patient isolates and the fact, which may be related, that only a small proportion of those infected develop clinical invasive amoebiasis. Mobile genetic elements, with their ability to alter gene expression may well be important in unravelling these puzzles.

## Background

Invasive amoebiasis (clinically manifested mainly as amoebic dysentery and amoebic liver abscess) remains one of the more significant human parasitic diseases, largely in less developed countries. The diseases is caused by infection with the protozoan *Entamoeba histolytica*, although it is now clear that only a minority of those infected will develop clinical amoebiasis [1]. All other species of *Entamoeba *that infect humans, and in particular the very closely related *E. dispar*, appear to be completely non-pathogenic [2,3].

The molecular biology of *E. histolytica *shows a number of unusual features, one of which is the abundance of polyadenylated but apparently untranslatable mRNAs produced [4]. Probably the most abundant of these transcripts are those first named IE [5,6]; the same sequences were subsequently described as *ehapt2 *[7] and *EhLSINE1*[8] but are now usually referred to simply as EhSINE1[9,10].

The genomic EhSINE1 sequences are known to be abundant, transcribed and polyadenylated but contain no plausible open reading frames. SINE elements (Short INterspersed repetitive Elements) are non-autonomous and are thought to use the enzymatic machinery of matching LINEs (Autonomous Non Long-Terminal-Repeat retroposons [Long INterspersed repetitive Element]) - in this case EhLINE1 [11]) for their transposition [12]. Importantly, this transposition mechanism results in the formation of flanking short direct repeats of the target site (Target Site Duplications, TSDs). Most EhSINE1s described so far are about 500-600 bp in length; this range is largely due to variable numbers of internal 26-27 bp repeats; the 5' and 3' terminal regions are normally well conserved. (For reviews of *E. histolytica *LINEs and SINEs see [9] and [10]).

SINEs are present in the genomes of many eukaryotes, and are particularly abundant in mammals [13] where they have had and continue to have far-reaching effects on gene expression. In *E. histolytica *irreversible transcriptional silencing of the gene coding for amoebapore (a pore-forming protein believed to be involved in pathogenicity) was achieved following transfection of parasites with a plasmid containing the regulating sequences of the amoebapore gene but also including the 5' segment of an adjacent EhSINE1 [14,15], although other work suggests that it was the fortuitous presence of two tRNA genes in the plasmid that is responsible for this unusual and little-understood phenomenon [16]. And finally the fact that the technique of Transposon Display involving EhSINE1 was successfully used to distinguish between four strains of *E. histolytica *[17] might suggest that these elements could have been recently mobile.

### Aims of this project

• Generation of EhSINE1 specific HMMs. As well as perfectly transposed EhSINE1s, truncated copies are usually present while even complete copies accumulate indels. This, plus the variable number of repeats make it difficult to compile a complete but non-redundant catalogue using BLAST searches. We therefore decide to develop an HMM model of EhSINE1 and use this to search for authentic copies in the newer JCVI-ESG2-1.0 reassembly of the *E. histolytica *genome.

• To analyse these EhSINE1s by length and internal repeat structure.

• To determine the length of the TSDs flanking each SINE. Newly transposed EhSINE1s will be flanked by TSDs, but over time these would be expected to decay, first becoming shorter and ultimately unrecognisable. The length of detectable TSDs might therefore be a rough measure of the time elapsed since transposition.

• To look for pairs or small groups of EhSINE1s that might represent recently transposed and could be used as a check on the use of TSD length to assess age.

• To examine SINE transcription by repeat class.

Here we report the identification of 393 EhSINE1s and divide them into classes based on the number of identifiable internal repeats. We show that the members of the separate classes differ in a number of ways which suggest that some are older than others and produce a small list of EhSINE1s which appear to have been recently transposed.

## Results & Discussion

### EhSINE1 inventory

Four hundred and eight potential EhSINE1s were initially identified; on examination 10 turned out to be EhSINE2s and 5 could not be convincingly recognised as SINEs at all. Three hundred and ninety three sequences were retained for analysis (Table 1 and Additional file 1). This number is similar to the 445 reported by Lorenzi *et al. *[10] using RepeatMasker and indeed with much older hybridisation data (approximately 500 copies per genome, [6]) but considerably more than the 272 found by Bakre *et al. *using BLAST [18].

**Table 1:** EhSINE1s detected in this study - basic summary data.

Repeats	No. of EhSINE1s. (%)	Length, range (bp)	Length , mean ± SD (bp)	With TSDs# - No. (%)		
				**All** **significant**	**No mis-match** **allowed**	**0 or 1 mis-** **match allowed**

1	158 (40)	451-536	510 ± 22.9*	131 (83)	53 (34)	80 (51)

2	67 (17)	543-555	548 ± 1.8	65 (97)	19 (29)	48 (71)

3	7 (2)	516-575	ND^§^	6 (86)	1 (14)	4 (57)

R1 only	6 (1)	467-539	ND^§^	5 (80)	1 (17)	3 (50)

R3 only	89 (23)	460-612	573 ± 27	78 (88)	13 (15)	48 (54)

4	3 (1)	600-602	ND^§^	3 (100)	0 (0)	3 (100)

None	63 (16)	451-684	561+58.7	42 (67)	19 (30)	33 (52)

TOTAL	393 (100)	451-684	541 ± 41.5	324 (82)	106 (27)	219 (56)

* Excludes those with 26 bp deletion

# By definition, ≥7 bp long

### Repeats

One hundred and fifty eight EhSINE1s had one repeat (R1; 1-rep SINEs), 67 two repeats (R1 and R2; 2-rep SINEs), 7 three repeats (R1, R2 and R3; 3-rep SINEs) and 3 four repeats (R1, R2, R3 and R4; 4-rep SINEs). In addition 95 EhSINE1s were of the appropriate length for 3-repeat ones but only the first (6 cases: "R1 only") or third (89 cases: "R3 only") repeat was recognizable. A further 63 EhSINE1s had no recognisable repeats at all (no-rep SINEs). No SINE1s with 5 or more repeats were identified. All repeats were similar in length (26 or 27 nt) and sequence (Additional file 2). A further five 1-repeat EhSINE1s were identified with the identical 26 bp deletion found in three of those discovered earlier.

### Insertion site preferences

EhSINE1s do not insert at specific recognition sites but have a preference for pyrimidine-rich regions [19]; Mandal *et al. *[11] have shown a strong preference for T-rich stretch of 15-20 nucleotides upstream of the insertion site. By comparing the base composition of the 50 bp region upstream and downstream of our EhSINE1s with the overall composition of the published genome, we confirmed this preference is for T-rich regions (Additional file 3). Overall 5' flanks averaged 54.9%T, and 3' flanks 51.7%T, compared with 37% for the genome as a whole. Broken down by repeat number 2-rep EhSINE1s had the greatest T-richness and 1-rep and no-rep the lowest; 5' flanks were always more T-rich than 3' but none of these differences reached statistical significance.

### Length and Sequence

**Figure 1:**
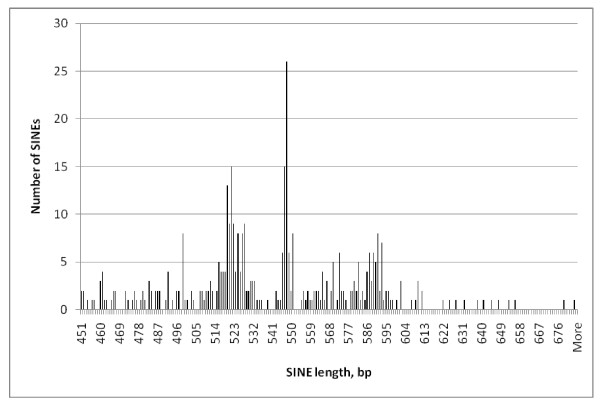
**Length histogram - all EhSINE1s**.

The lengths of the 393 SINE1s ranged from 451- 684 bp but were not normally distributed; instead their peak numbers largely coinciding with the expected lengths of 1, 2 3 or 4-rep EhSINE1s (Figure 1). Those which did not cluster around one of these four expected lengths were checked individually; in all cases the discrepancy could be explained by unique insertions or deletions.

**Figure 2:**
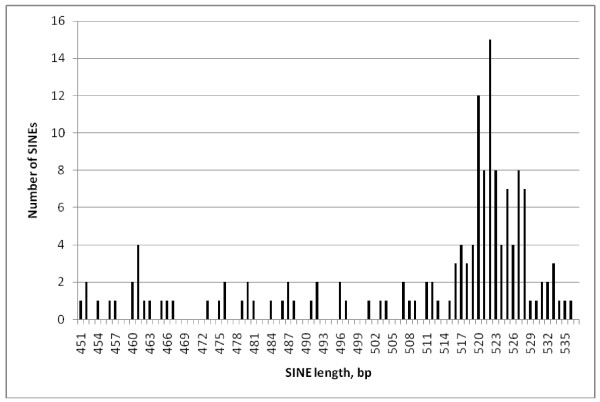
**Length histogram - 1-rep EhSINE1s**.

**Figure 3:**
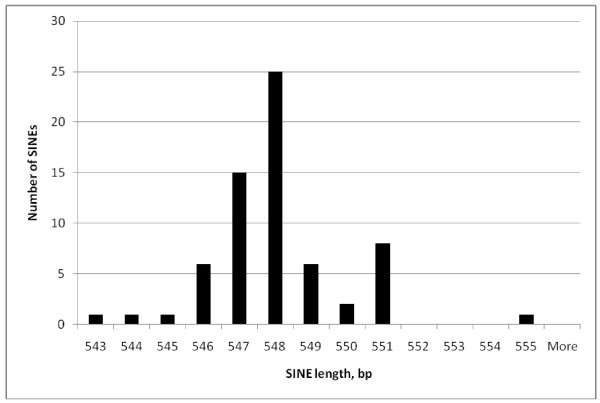
**Length histogram - 2-rep EhSINE1s**.

**Figure 4:**
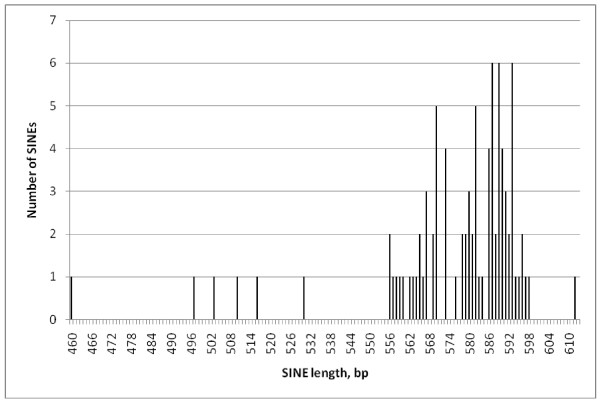
**Length histogram - R3-only EhSINE1s**.

**Figure 5:**
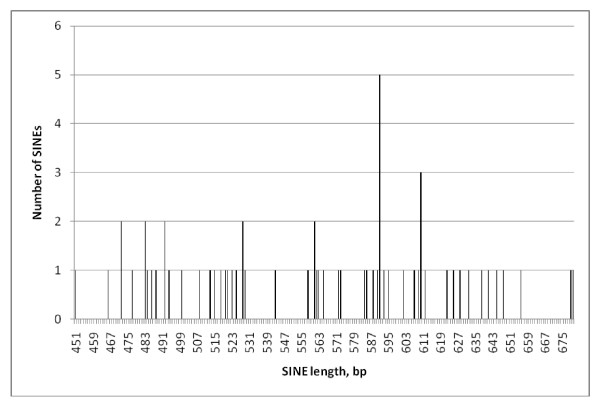
**Length histogram - no-rep EhSINE1s**.

Comparing the lengths of individual repeat classes (Figures 2, 3, 4 and 5) EhSINE1s with 2 repeats (2-reps) were very tightly clustered in contrast to 1-rep and (R3 only) EhSINE1s which showed a clear peak but a long tail of shorter sequences. No-rep EhSINE1s had a broad length range. There were too few 3-rep, R1 only and 4-rep EhSINE1s to analyse their length variation.

**Figure 6:**
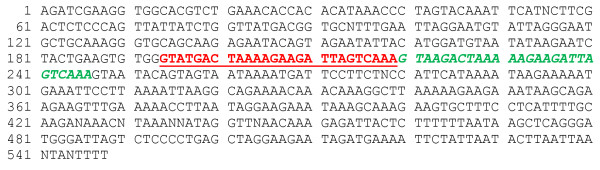
**60% consensus sequence of the 65 2-rep EhSINE1s identified in this study**. Bold, underlined, red - R1; bold, italic, green - R2.

**Figure 7:**
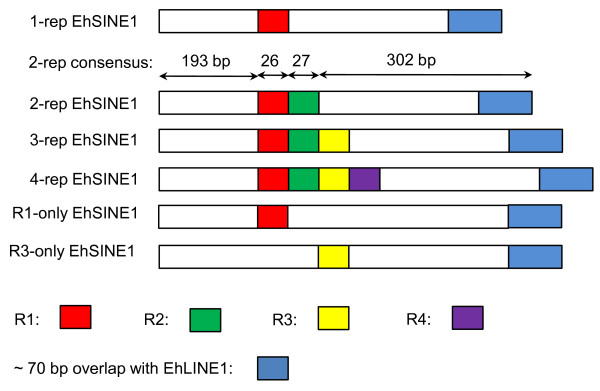
**Map of the EhSINE1 classes (excluding those with no repeats)**. Lengths given are for the consensus sequence of the 65 2-rep EhSINE1s - the most homogeneous class.

The 67 2-rep EhSINE1s are highly homogeneous; a 60% consensus sequence is shown in Figure 6. A map of the various EhSINE1 classes is shown in Figure 7; it also shows the approximately 70 bp overlap with EhLINE1 first identified by Van Dellen *et al. *[8] and believed to be the mechanism whereby SINEs are transposed by their partner LINEs.

The origin of these internal repeats is not clear but it is possible to imagine that either unequal crossing over or strand slippage leads to the replication of two copies of virtually identical sequence, which can then expand via strand slippage etc. The *de novo *origins of repeated sequences are may not be fully understood but the complex patterns of such repeats found between the tRNA genes of *E. histolytica *[20] confirm that they can arise in this organism.

Assuming that *E. histolytica *SINEs are not under selective pressure it is plausible to imagine that newly transposed EhSINE1s will decay over time because of insertions or deletions to the point where the repeats become unrecognisable; this would suggest that 2-rep EhSINE1s are the result of the most recent wave of transposition and those with no recognisable repeat possibly the oldest.

### TSD length by repeat class

In all, 324 EhSINE1s (82% of the total) had a clearly recognisable TSD (Table 1). Analysed by repeat class, 83% of 1-rep EhSINE1s have a recognisable TSD, length centred about 9-13 bp; almost all (65/67) 2-rep EhSINE1s have TSDs and these are much longer, most being 18-24 bp long (the sequences of the two without TDRs were carefully checked and found to be quite typical 2-rep EhSINE1s). The number of R1 only, 3-rep and 4-rep EhSINE1s was too small for analysis but 88% of R3-only EhSINE1s had TSDs, the majority around 11-13 bp long. Finally, 67% of EhSINE1s without recognisable repeats had TDRs but these were shorter, most being between 6-12 bp long (Additional file 4).

We also repeated the analysis for TSDs with zero or 1 mismatch allowed. The number of TSDs identified is now much smaller (106 and 219 respectively) but the same variation in TSD length with repeat class is still visible as long as at least one mismatch is allowed (the numbers in each bin if no mismatches are permitted are too small for analysis).

Because of the way in which they are mobilised by their partner LINEs, newly transposed EhSINE1s will be flanked by TSDs [11], there is currently no reason to believe that these TSDs will not be of random length. But over time the two TSDs would be expected to suffer (different) random mutations, with identical stretches first becoming shorter and ultimately unrecognisable as duplicated sequences. Our hypothesis, therefore is that long TSDs might indicate recent transposition again suggesting that most 2-rep EhSINE1s might be the relatively young.

### Recently transposed SINE1s

We identified 19 sets of "identical" (99 or 100% sequence identity) EhSINE1s - 14 pairs and one set each containing 3, 4, 5 and 6 members. Of these sets, only eight (7 pairs and 1 triplet) showed no similarity in their flanking sequences, and are thus prime candidates for having been recently transposed (Table 2), six (including all the sets with more than three members) had identical flanks and are regarded as assembly errors or large-scale duplications, which have been detected in the new assembly (Lorenzi, personal communication) and four were ambiguous - mainly owing to very little flanking sequence being available.

**Table 2:** Pairs (and one triplet) of possibly recently-transposed EhSINE1s.

Set	SINE	Location	Length (bp)	**Or**.	Repeats	TSD length (bp)
**A**	**EhSINE1_1**	**gi|169801175|gb|DS571347.1| 7763-8309**	**547**	**+**	**2**	**29**

**A**	**EhSINE1_2**	**gi|169801175|gb|DS571347.1| 2150-2696**	**547**	**+**	**2**	**11**

B	EhSINE1_15	gi|169802311|gb|DS571190.1| 67757-68303	547	+	2	21

B	EhSINE1_6	gi|169800595|gb|DS571494.1| 2811-3357	547	+	2	29

B*	EhSINE1_230	gi|169803070|gb|DS571147.1| 112278-111732	547	-	2	25

E*	**EhSINE1_206**	**gi|169800562|gb|DS571508.1| 4500-3954**	**547**	**-**	**2**	**21**

**E**	**EhSINE1_18**	**gi|169801330|gb|DS571317.1| 27553-28099**	**547**	**+**	**2**	**24**

F*	EhSINE1_207	gi|169801573|gb|DS571277.1| 39116-38571	546	-	2	24

F	EhSINE1_34	gi|169799366|gb|DS572252.1| 2-548	547	+	2	N/A#

**G**	**EhSINE15_42**	**gi|169800336|gb|DS571597.1| 8573-9089**	**517**	**+**	**1**	**26**

G*	**EhSINE1_248**	**gi|169800938|gb|DS571399.1| 19037-18521**	**517**	**-**	**1**	**16**

J	EhSINE1_149	gi|169801894|gb|DS571234.1| 34641-35201	561	+	None	8

J	EhSINE1_341	gi|169801894|gb|DS571234.1| 29270-28710	561	-	None	0

O*	**EhSINE1_224**	**gi|169800830|gb|DS571423.1| 1030-483**	**548**	**+**	**2**	**13**

**O**	**EhSINE1_219**	**gi|169798957|gb|DS572593.1| 697-150**	**548**	**+**	**2**	**24**

P	EhSINE1_232	gi|169801851|gb|DS571240.1| 35235-34688	548	+	2	15

P*	EhSINE1_236	gi|169801851|gb|DS571240.1| 22360-21813	548	+	2	21

* 99% identical to other member(s) of the set; otherwise 100%

# Not available; no flanking sequence available at 5' end to assess TSD length. But this pair qualifies for inclusion as the 3' flanks are not similar

Of the eight remaining sets, the two EhSINE1s in set J, although meeting the criteria of having non-identical flanks, are anomalous in having a rather divergent sequence and being the only ones having no significant hits in the cDNA library (see below) and were also excluded.

If the remaining 15 EhSINE1s are genuinely young (rather than duplications or mis-assemblies) there is no reason to expect that their TSDs would be similar in length, and this is true, the average difference in TSD length in the seven pairs being 8.6 bp. If long TSDs are a mark of a young SINE we would expect the 15 "genuinely recent" EhSINE1s to have longer TSDs than a random SINE and this is also seen: 21 ± 5 bp *v *13 ± 8 bp for the whole set. And if 2-rep EhSINE1s are the youngest we would expect them to be over-represented - true: 13/15 (87%) *v *67/393 (17%) of all EhSINE1s. So all these measures are consistent with a small number of EhSINE1s having been transposed recently although, of course, the data is very sparse.

If EhSINE1s have indeed been mobile in the recent past it is natural to wonder if they still are today. As they are nonautonomous, mobility will require the presence of a functional LINE1 element; although Lorenzi *et al. *identified 88 complete Eh_LINE1s they all contained stop codons or frame shifts in a least one of their putative ORFs [10]. However they consider it possible that the defective protein from one LINE could be replaced by that from another with a mutation in a different ORF; a similar complementation in *trans *has been demonstrated experimentally in *Drosophila *[21].

### Transcription

EhSINE1s are extensively transcribed - they were the second most abundant transcript in a small *E. histolytica *cDNA library [7]. To explore this further we carried out a BLAST search of our EhSINE1 set against a set of 19901 *E. histolytica *cDNAs in GenBank. Because of the high similarity between all SINE1 sequences, only cDNAs which met the criteria of having an e-value of 0.0, Identity of at least 90% and which covered at least 90% of the SINE length were scored as unequivocally genuine SINE transcripts - 142 such were detected (Additional file 5).

When examined by repeat length interesting differences emerge - while 94% of 2-rep EhSINE1s appear to be transcribed, only 45% of 1-rep and about 1% of R3-only and no-rep EhSINE1s are. Other groups are too small for analysis but it is interesting that all three of the long, 4-rep EhSINE1s have unique transcripts which meet our criteria. It may also be significant that all of the of transcripts from the 2-rep EhSINE1s are virtually full-length (Additional file 6).

### Promoters

Most eukaryotic SINEs are derived from small RNA molecules which are normally transcribed by Pol III. *E. histolytica *SINE1s are unusual in that no precursor small RNA has been identified as yet, and they do not fit into any of the groups proposed by Wicker *et al. *[22]. But hypothesising that they are nevertheless transcribed by Pol III we looked to see if recognisable internal promoters could be detected.

Pol III recognises two motifs, the A box and the B box [23]. The consensus sequences of both well and poorly transcribed classes of SINEs contain a motif (TcG--AAgGTGGC) which is similar but not identical to the canonical A box (TGGCNNAGTGGN). The consensus sequence of all 2-rep EhSINE1s contain a sequence (ncTTCGActCTCC)which again shows some similarity to a B box (GGTTCGANNCC), and this may be adequate to promote transcription by Pol III.

The consensus sequences of both poorly transcribed SINE1 families have "B boxes" which are less similar to the canonical sequence (R3 only: GtTTtaaCTCt; no-rep: atTTCaAgttttgaatCACC) but whether this difference is significant is not clear at present.

*E. histolytica *tRNA genes are presumably transcribed by Pol III. The majority of these genes are arranged in unique arrays [20]; Dr Graham Clark kindly supplied us with the sequences of all these genes, from which, after aligning them, motifs very similar to the A box, and identical to the canonical B box could be identified (TaGCTCAGTtGGT and GGTTCGATCCC) - suggesting that *E. histolytica *Pol III recognises typical eukaryotic internal promoters. An apparent A box with the sequence AGATTAGCATGG has also been annotated in the *E. histolytica *U6 snRNA gene [24].

### Are they SINEs or degenerate LINEs?

Nikaido and Okada proposed that a SINE should be defined by the presence of a region homologous to a tRNA or to 7SL RNA, together with A-box and B-box promoter sequences [25]; by this definition our EhSINE1s are not SINEs. They went on to show that the cetartiodactyl sequences ARE1p, ARE2p, CetSINE1, and CetSINE2 are not SINEs but are, in fact, only partial sequences of members of a new subfamily of LINEs. Going further, Kramerov and Vassetzky [13] claimed that there are no SINEs in protozoa, they are all fragments of autonomous elements. To at least partially test this we prepared a truncated consensus 2-repeat SINE by removing 100 bp at 3' end (which is known to be shared by the partner LINE [8]) and compared it to a reconstructed sequence of EhLINE1 (kindly provided by Prof Sudha Bhattacharya). No significant similarity was found.

Non-autonomous retrotransposons of a similar size to EhSINE1 have been extensively studied in trypanosomatids, where LTR retrotransposons and site-specific and non site-specific retrotransposons (but no DNA transposons) are present [26]. The latter group contain two pairs - a large, apparently (once) autonomous retrotransposon and a small non-autonomous one that presumably relied on it for mobilisation - that suggest similarities to the *E. histolytica *EhLINE1/EhSINE1 pair. However, in both cases (*ingi*/RIME in *Trypanosoma brucei *[27,28] and LiTc/NARTc in *T. cruzi *[29,30]) the small non-autonomous element shares significant sequence similarity along its whole length (as well as a highly conserved 45 bp motif at the 5' end) with its partner autonomous retrotransposon. This is significantly different from the EhLINE1/EhSINE1 case where only approx 70 bp at the 3'end are shared, and suggests that in the trypanosomatid case, the small nonautonomous elements were also derived from the autonomous ones by 3' deletion [26].

Three LINE-like non-LTR retrotransposons have been identified in the genome of *Giardia intestinalis *(*G. lamblia*) but no other classes of mobile genetic elements [31]. Transposable elements make up a very significant fraction of the genome of *Trichomonas vaginalis *but the vast majority are DNA transposons with only two, large, retrotransposons reported [32].

Since the subject of mobile genetic elements of protozoan parasites was reviewed eight years ago [33] only a modest number of new ones have been reported. However work such as that of Lorenzi *et al. *[10] suggest that the rapid increase in the number of protozoan genomes sequenced, coupled with intensive examination of that data, will soon reveal many more.

## Conclusions

By using a Hidden Markov Model to search the newly assembled *E. histolytica *genome we have identified 393 copies of a SINE element - EhSINE1; this number is in reasonable agreement with that found by Lorenzi *et al. *and with older hybridisation data. Examination of the internal structure of these EhSINE1s reveals 1-4 copies of a 26-27 nt repeat in many, together with other EhSINE1s in which fewer repeats than expected for the length of the SINE, or no repeats at all, can be detected.

Our working hypothesis was that newly transposed EhSINE1s would be identical in sequence to the parent copy and would have easily-recognisable TSDs, but that over time these similarities would decay, providing a rough measure of time since transposition.

Our evidence suggests that EhSINE1s with two repeats (2-rep EhSINE1s) do indeed show the properties expected of relatively recently transposed specimens - they are the most homogenous group, with the smallest range of lengths and they tend to have longer TSDs. They are also most likely to have matching cDNAs in an *E. histolytica *cDNA library.

Because EhSINE1s insert randomly (although with a strong preference for T-rich sites) they presumably need to rely on internal promoters if they are to be transcribed, and again it is reasonable to suppose that over times these promoter sequences will decay and become non-functional. (The enzyme responsible for transcribing these EhSINE1s is not known but would be expected to be Pol III, although we could only identify sequences with limited similarity to the canonical A and B boxes) so the fact that 2-rep EhSINE1s appear to be the most likely to be transcribed again supports their fairly recent mobility.

SINEs are non-autonomous and require matching LINEs if they are to transpose. Although the matching EhLINE1 is well known, there are no known copies free from stop codons or frame shifts in one or other of their two open reading frames. Nevertheless the final stages of this decay might have been very recent and/or proteins from separate ORFs on two different LINEs might co-operate so that recent or present day mobility is not impossible. We specifically looked for pairs of EhSINE1s which might provide evidence of this by having identical or very nearly identical sequences but different flanking sequences (to try to exclude mis-assemblies or large-scale duplications) and identified 15 such (6 pairs and one triplet). Interestingly, although the small numbers make formal analysis impossible, 13/15 of these were 2-rep EhSINE1s.

A number of recent papers, particularly those of Bakre *et al. *and Lorenzi *et al. *[10,18] have surveyed the whole range of repetitive elements which are now known to make up nearly 20% of the *E. histolytica *genome. Here we have looked in more detail at one member of this group of elements and shown that it is possible to detect sub-populations that seem to have arisen from waves of transposition at different times in the past, and also shown that while present-day mobility cannot be demonstrated there is no evidence that it is not still occurring to some extent.

We are currently working to improve our ability to recognise short TSDs in the very T-rich *E. histolytica *genome, and to determine a probability measure that they have not arisen by chance; related to this we wish to model the decay of originally long repeats through random mutation. We also intend to investigate the wider questions of how often SINE insertions have disrupted functional genes, whether this process really is continuing today and, ultimately, if it has any bearing on the outcome of infection with this organism.

## Methods

### Generation of Hidden Markov Models for EhSINE1 identification

Based on earlier unpublished work 50 authentic EhSINE1 sequences had been identified by BLAST searches [34] of the original eha1 *E. histolytica *genome assembly http://www.tigr.org/tdb/e2k1/eha1/[35] and each one examined in detail to determine the number of repeats present. They comprised 9 with 1 repeat (3 with a specific and identical 26 bp deletion), 33 with 2 repeats, 5 with 3 and 3 with 4. No EhSINE1s with more than 4 repeats could be identified despite intensive searches. These four groups of EhSINE1s were then aligned using CLUSTAL W version 2 (CLUSTAL) [36] and 30% consensus sequences obtained using SEAVIEW [37].

**Figure 8:**
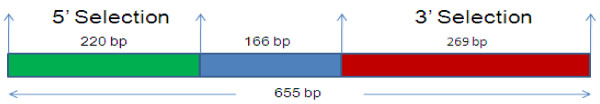
**Schematic diagram of the 5' and 3' sections of the first EhSINE1 consensus sequence used to prepare the Hidden Markov Models**.

The consensus sequences were used to prepare an HMM of EhSINE1. The consensus sequences were first aligned with CLUSTAL and the 5' and 3' sections of the alignment (see Figure 8) separated out into individual files from which HMM models of each were created with HMMER http://hmmer.janelia.org. The newer JCVI-ESG2-1.0 reassembly of the *E. histolytica *genome (GenBank WGS_SCAFLD NW_001914860-NW_001916388) was downloaded and searched with these HMMs Putative EhSINE1s were identified using custom Perl scripts to parse the HMMER output and identify each predicted 5' and 3' region where the overall length, including intervening sequence, was not greater than 700 bp. Using individual HMM files for the 5' and 3' regions enabled the identification of all EhSINE1s, irrespective of the number of internal repeats.

Each hit was aligned with an overall consensus EhSINE1 to determine, by comparison with the highly conserved 5' and 3' approximately 100 bp sequences, if it was an authentic SINE1. If so, it was then aligned with as many of the consensus sequences as necessary to determine the number of recognisable repeats.

### Repeat consensus sequences

Authentic SINE1s were thus divided into those with 1, 2, 3 & 4 repeats, no recognisable repeats and those which, although the correct length for 3-repeat EhSINE1s had only R1 or R3 recognisable.

All EhSINE1s in each repeat class were then aligned using CLUSTAL, repeats identified, extracted and a 60% consensus sequence of the repeats prepared using SEAVIEW. The 2-rep EhSINE1s are sufficiently homogeneous for a 60% consensus sequence of the whole SINE to be prepared in a similar manner. These sequences have been submitted to RepBase http://www.girinst.org/repbase.

### T-richness of flanking regions

Fifty bp sequences 5' and 3' to all EhSINE1s were extracted. In six cases these consisted entirely or largely of "N"s, these were discarded as were a small number of very short sequences where the SINE was very close to the end of an assembly. Percentage of Ts were calculated for the 5' and 3' flanks as a whole and also sorted by repeat class.

### TSDs

TSDs were identified by isolating 50 bp flanking regions from each EhSINE1 and aligning them using the BLAST bl2seq program. The output was parsed with a custom Perl script and TSDs identified. EhSINE1s preferentially insert into very T-rich regions of the *E. histolytica *genome which makes short TSDs impossible to recognise convincingly - any hits ≤6 bp long were not significant and were scored as zero (no TSD).

### Possible recently transposed EhSINE1s

"Identical" EhSINE1s were detected by preparing a FASTA database of all 393 validated SINE1s and carrying out an all against all BLAST search. 100 and 99% identical sequences were selected and assembled into non-redundant pairs or small groups. The sequence of each pair of EhSINE1s plus 1000 bp of 5' and 3' flanking sequence (or as much as possible, when the SINE was close to the end of an assembly) was then extracted and compared using Dotter [38]. Pairs in which the flanking sequence was identical were excluded as probable assembly errors, as were those where very little flanking sequence was available.

### Transcription

Evidence for transcription was sought by carrying out a BLAST search of our EhSINE1 set against a set of 19901 *E. histolytica *cDNAs in GenBank (accession numbers CX079516-CX099417). Only cDNAs which met the criteria of having an e-value of 0.0, Identity of at least 90% and which covered at least 90% of the SINE length were scored as genuine SINE transcripts. We examined in detail the 63 cDNAs which by our criteria were genuine transcripts from 2-rep EhSINE1s. Removing redundant hits (where the same cDNA was the closest match to more than one EhSINE1) we were left with 41 unique cDNAs. Four long cDNAs (gi|56625926, gi|56596210, gi|56597072 and gi|56598670) were the only ones in the opposite orientation to our SINE sequence and may be the result of transcription of a protein-coding gene with a downstream EhSINE1 running on. One (gi|56593923) was a long transcript from a 4-rep SINE and another (gi|56599038) a short one from a 1-rep SINE. The remaining 35 cDNA sequences were aligned (CLUSTAL) with the 2-rep consensus sequences (Additional file 6).

## Authors' contributions

DH and JA jointly designed and carried out the study. DH prepared the HMM model, wrote the Perl scripts and carried out the data extraction and computational work. JA examined and analysed the SINE sequence sets and wrote the first draft of the paper. IP carried out the initial work on the TSD-finding algorithms; SB participated in the design and coordination of the project.

All authors have read and approved the final version of the manuscript.

## Supplementary Material

Additional file 1 **All EhSINE1 data**. Full details of all the EhSINE1s identified in this study. Click here for file

Additional file 2 **Repeat consensus sequences**. Consensus sequences of R1, R2, R3, R4 EhSINE1 repeats. Click here for file

Additional file 3 **T-richness of flanking sequences**. T-richness of 5' and 3' 50 bp flanks of EhSINE1s found in this study. Click here for file

Additional file 4 **TSD lengths by repeat class**. Length histograms of TSDs for major classes of EhSINE1s identified in this study. Click here for file

Additional file 5 **EhSINE1 transcription data**. Transcript (cDNA) BLAST hits for all EhSINE1s identified in this study. Cells are colour coded; white ones have an E-value of 0, ID of at least 90% and cover at least 90% of the SINE and are the only ones considered for inclusion as genuine transcripts in this study (see Methods for details). Blue, red and yellow cells indicate progressively less impressive hits. Click here for file

Additional file 6 **Alignment of 35 *E. histolytica *cDNAs with the 2-rep EhSINE1 consensus sequence**. Demonstrates that most 2-rep EhSINE1 transcripts are almost full-length. Click here for file
