# Supplementary material for: Bioinformatic analysis of Entamoeba histolytica SINE1 elements
Source: BMC Genomics. 2010 May 24;11:321. doi: 10.1186/1471-2164-11-321 (PMC2996970; doi:10.1186/1471-2164-11-321)
Supplement: Additional file 2 — Repeat consensus sequences. Consensus sequences of R1, R2, R3, R4 EhSINE1 repeats. [file 1471-2164-11-321-S2.DOC]

## Supplemental Table 2 – Repeat consensus sequences

R1 consensus sequence from 148 1-repeat EhSINE1s

GTAAGACTAAAAGAAAAATTAAACATA

R1 consensus sequence from 67 2-repeat EhSINE1s

GTATGACTAAAAGAAGATTAGTCAAA

R1 consensus sequence from 7 3-repeat EhSINE1s

GTATGACTAAAAAGAAGATTAGTCAAA

R1 consensus sequence from 3 4-repeat EhSINE1s

GTATGACTAAAAGAAGATTAGTCAAA

R2consensus sequence from 67 2-repeat EhSINE1s

GTAAGACTAAAAAGAAGATTAGTCAAA

R2 consensus sequence from 7 3-repeat EhSINE1s

GNAAGACTAAAAAGAAGATTAGTCAAA

R2 consensus sequence from 3 4-repeat EhSINE1s

GTAAGACTAAAAAGAAGATTAGTCAAA

R3 consensus sequence from 7 3-repeat EhSINE1s

GNAAGACTAAAAAGAAGATTAGTCAAA

R3 consensus sequence from 3 4-repeat EhSINE1s

GTAAGACTAAAAAGAAGATTAGTCAAA

R3_ consensus sequence from 89 R3-only EhSINE1s

GTAAGACTAAAAGGAAAATTAATCATA

R4 consensus sequence from 3 4-repeat EhSINE1s

GTAAGACTAAAAAGAAGATTAGTCAAA

Consensus sequences of EhSINE1 repeats
