# Supplementary material for: Bioinformatic analysis of Entamoeba histolytica SINE1 elements
Source: BMC Genomics. 2010 May 24;11:321. doi: 10.1186/1471-2164-11-321 (PMC2996970; doi:10.1186/1471-2164-11-321)
Supplement: Additional file 3 — T-richness of flanking sequences. T-richness of 5' and 3' 50 bp flanks of EhSINE1s found in this study. [file 1471-2164-11-321-S3.DOC]

**Supplemental Table 3: T-richness of flanking sequences**

| **%T** | **1 rep 5’** | **1 rep 3’** | **2 rep 5’** | **2 rep 3’** | **All 3 rep 5’** | **All 3 rep 3’** | **All no rep 3’** | **All no rep 3’** | **All 5’** | **All 3’** |
| --- | --- | --- | --- | --- | --- | --- | --- | --- | --- | --- |
| Average | 54.9 | 50.5 | 57.1 | 52 | 55.2 | 55.5 | 52.2 | 48 | 54.9 | 51.7 |
| SD | 7.8 | 11.4 | 9 | 10.8 | 8.4 | 12 | 12.7 | 14.2 | 9.2 | 12.2 |

T-richness of 5’ and 3’ 50bp flanks of SINEs found in this study. The overall T-richness of the *E. histolytica* genome is 37%
