# Supplementary material for: Bioinformatic analysis of Entamoeba histolytica SINE1 elements
Source: BMC Genomics. 2010 May 24;11:321. doi: 10.1186/1471-2164-11-321 (PMC2996970; doi:10.1186/1471-2164-11-321)

## Additional file 4: TSD lengths

## Supplemental Figure 1 – TSD lengths of 1-rep SINEs


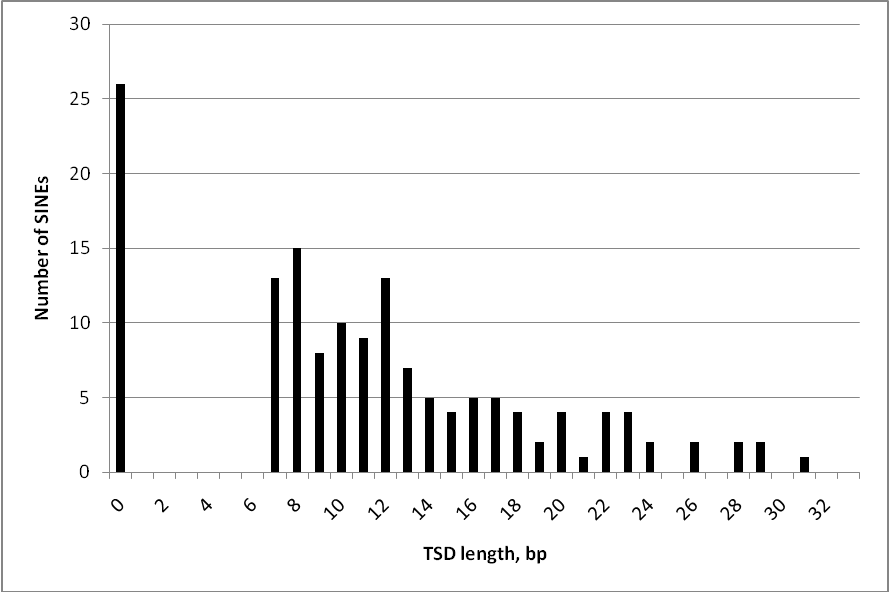


## Supplemental Figure 2 – TSD lengths of 2-rep SINEs


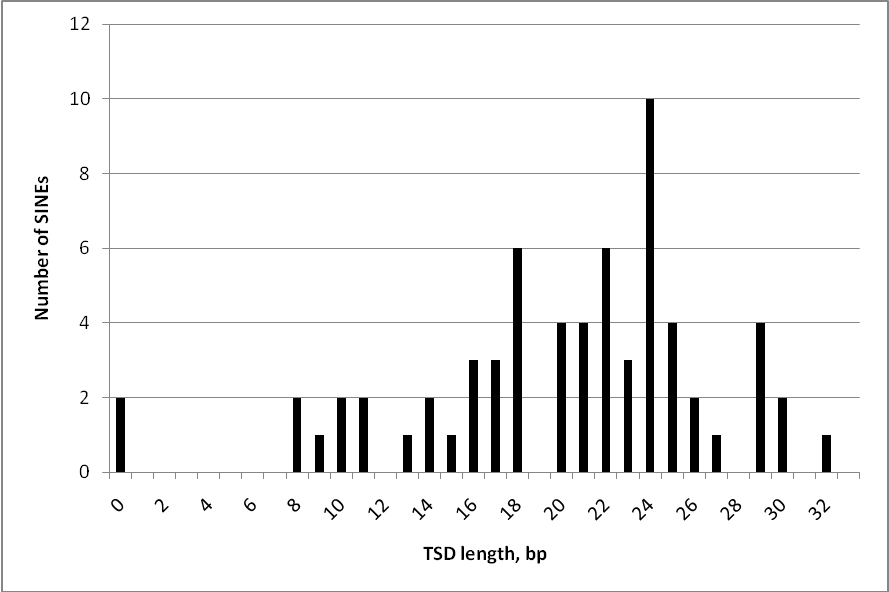


## Supplemental Figure 3 – TSD lengths, R3-only SINEs


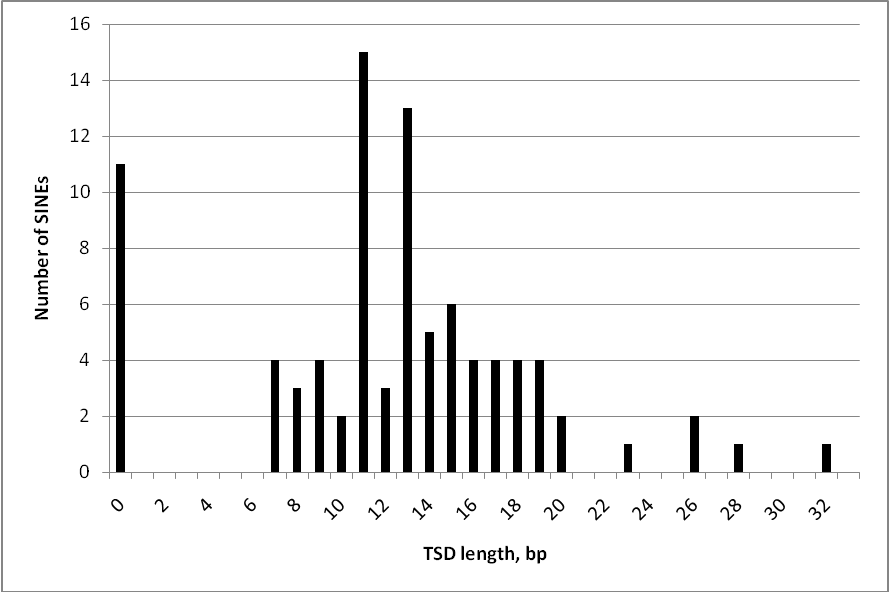


## Supplemental Figure 4 – TSD lengths, None-rep SINEs


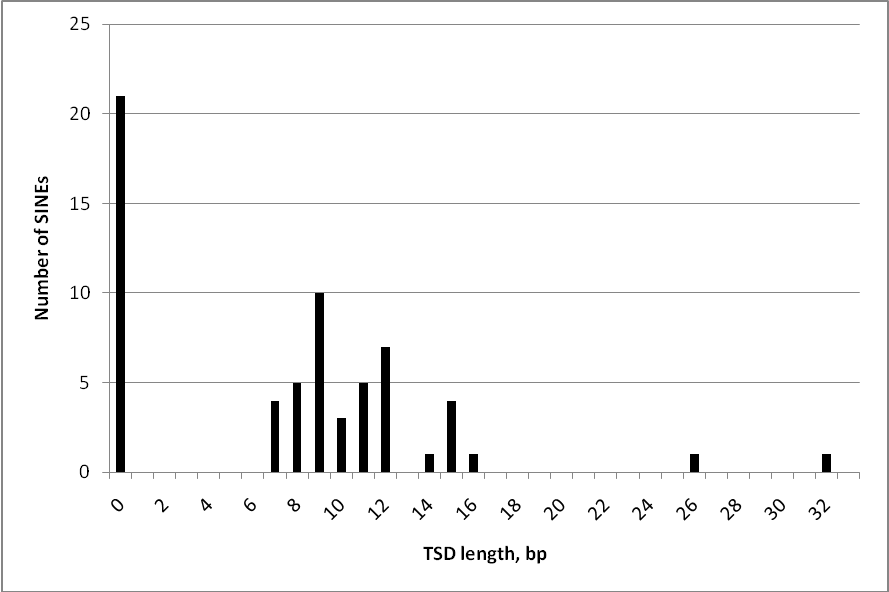

Supplement: Additional file 4 — TSD lengths by repeat class. Length histograms of TSDs for major classes of EhSINE1s identified in this study. [file 1471-2164-11-321-S4.DOC]
