# Supplementary material for: Bioinformatic analysis of Entamoeba histolytica SINE1 elements
Source: BMC Genomics. 2010 May 24;11:321. doi: 10.1186/1471-2164-11-321 (PMC2996970; doi:10.1186/1471-2164-11-321)
Supplement: Additional file 6 — Alignment of 35 E. histolytica cDNAs with the 2-rep EhSINE1 consensus sequence. Demonstrates that most 2-rep EhSINE1 transcripts are almost full-length. [file 1471-2164-11-321-S6.DOC]

## Supplemental Figure 5

Consensus_65_2-rep_EhSINE1s -----------AGATCGAAGGTGGCACGTCTGAAACACCACACATAAACC

gi|56594149|gb|CX080159.1|CX08 ---------------------------GTCTGAAACACCACACATAAACC

gi|56594501|gb|CX080511.1|CX08 ------------------------CACATCTAAAACACCACACATAAACC

gi|56594583|gb|CX080593.1|CX08 --------------------GTGGCACGTCTGAAACACCACACATAAACC

gi|56594699|gb|CX080709.1|CX08 ------------------------CACGTCTGAAACACCACACATAAACC

gi|56595210|gb|CX081220.1|CX08 ---------------------------GTCTGAAACACCACACACAAACC

gi|56595226|gb|CX081236.1|CX08 ------------------------CACGTCTGAAACACCACACACAAACC

gi|56595284|gb|CX081294.1|CX08 ------------------------CACGTCTGAAACACCACACACAAACC

gi|56595554|gb|CX081564.1|CX08 ------------------------CACGTCTGAAACACCACACATAAACC

gi|56595866|gb|CX081876.1|CX08 -----------------AAGGTGGCACGTCTGAAACACCACACACAAACC

gi|56596052|gb|CX082062.1|CX08 ---------------------------GTCTGAAACACCACACATAAACC

gi|56596773|gb|CX082783.1|CX08 -------------------------TCGTCTGAAACACCACACATAAACC

gi|56596919|gb|CX082929.1|CX08 ---------------------------GTCTGAAACACCACACACAAACC

gi|56596986|gb|CX082996.1|CX08 ---------------------GATCACGTCTGAAACACCACACATAAACC

gi|56597956|gb|CX083966.1|CX08 --------------------GTGGCACGTCTGAAACACCACACATAAACC

gi|56598330|gb|CX084340.1|CX08 --------------------GTGGCACGTCTGAAACACCACACATAAACC

gi|56599621|gb|CX085631.1|CX08 -------------------GGTGGCACGTCTGAAACACCACACACAAACC

gi|56601669|gb|CX087675.1|CX08 ------------------------CACGTCTGAAACACCACACATAAACC

gi|56602105|gb|CX088111.1|CX08 ------------------------CACGTCTGAAACACCACACATAAACC

gi|56602188|gb|CX088194.1|CX08 ---------------------------GTCTGAAACACCACACACAAACA

gi|56602405|gb|CX088411.1|CX08 ------------------------CACGTCTGAAACACCACACATAAACC

gi|56602598|gb|CX088604.1|CX08 ------------------------CACGTCTGAAACACCACACACAAACC

gi|56602608|gb|CX088614.1|CX08 ---------------------------GTCTGAAACACCACACACAAACC

gi|56603426|gb|CX089432.1|CX08 --------------------------TTCCTGAAACACCACACATAAACC

gi|56615281|gb|CX090913.1|CX09 -------------------GGTGGCACGTCTGAAACACCACACATAAACC

gi|56616332|gb|CX091964.1|CX09 -----------------------TCACGTCTGAAACACCACACACAAACC

gi|56617204|gb|CX092522.1|CX09 ------------------------CACGTCTGAAACACCACACGCAAACA

gi|56617980|gb|CX092894.1|CX09 -----------------------GTACGTCTGAAACACCACACATAAACC

gi|56620877|gb|CX094380.1|CX09 ------------------------CACGTCTGAAACACCACACATAAACC

gi|56622641|gb|CX095382.1|CX09 GTTATTATCTGGTTATGACGGTGGCACGTCTGAAACACCACACGCAAACA

gi|56623152|gb|CX095893.1|CX09 ------------------------CACGTCTGAAACACCACACATAAACC

gi|56625177|gb|CX097763.1|CX09 --------------------------CGTCTGAAACACCACACATAAACC

gi|56626119|gb|CX098234.1|CX09 -------------------GGTGGCACGTCTGAAACACCACACACAAACC

gi|56627180|gb|CX098742.1|CX09 -------------------GGTGGCACGTCTGAAACACCACACACAAACC

gi|56627915|gb|CX099120.1|CX09 ------------------------CACGTCTGAAACACCACACATAAACC

gi|56628353|gb|CX099338.1|CX09 ---------------------------GTCTGAAACACCACACATAAACC

** *********** ****

Consensus_65_2-rep_EhSINE1s CTAGTACAAATT-CATNC--TTCGACT-CTCCCAGTTATTATCTGGTTAT

gi|56594149|gb|CX080159.1|CX08 CTAGTACAAATT-CATTC--TTCGACT-CTCCCAGTTATTATCTGGTTAT

gi|56594501|gb|CX080511.1|CX08 CTAGTACAAATT-CATTC--TTCGACT-CTCCCAGTTATTATCTGGTTAT

gi|56594583|gb|CX080593.1|CX08 CTAGTACAAATT-CATAC--TTCGACT-CTCCCAGTTACTATCTGGTTAT

gi|56594699|gb|CX080709.1|CX08 CTAGTACAAATT-CATTC--TTCGACT-CTCCCAGTTATTATCTGGTTAT

gi|56595210|gb|CX081220.1|CX08 CTAGTACAAATT-CAAAC--TTCGACT-CTCCCAGTTATTATCTGGTTAT

gi|56595226|gb|CX081236.1|CX08 CTAGTACAAATT-CATTC--TTCGACT-CTCCCAGTTATTATCTGGTTAT

gi|56595284|gb|CX081294.1|CX08 CTAGTACAAATT-CATTC--TTCGACT-CTCCCAGTTATTATCTGGTTAT

gi|56595554|gb|CX081564.1|CX08 CTAGTACAAATT-CATTC--TTCGACT-CTCCCAGTTATTATCTGGTTAT

gi|56595866|gb|CX081876.1|CX08 CTAGTACAAATT-CATTC--TTCGACT-CTCCCAGTTATTATCTGGTTAT

gi|56596052|gb|CX082062.1|CX08 CTAGTACAAATT-CATTC--TTCGACT-CTCCCAGTTATTATCTGGTTAT

gi|56596773|gb|CX082783.1|CX08 CTAGTACAAATT-CATAC--TTCGACT-CTCCCAGTTATTATCTGGTTAT

gi|56596919|gb|CX082929.1|CX08 CTAGTACAAATT-CATTC--TTCGACT-CTCCCAGTTATTATCTGGTTAT

gi|56596986|gb|CX082996.1|CX08 CTAGTACAAATT-CATTC--TTCGACT-CTCCCAGTTATTATCTGGTTAT

gi|56597956|gb|CX083966.1|CX08 CTAGTACAAATT-CATTC--TTCGACT-CTCCCAGTTATTATCTGGTTAT

gi|56598330|gb|CX084340.1|CX08 CTAGTACAAATT-CATTC--TTCGACT-CTCCCAGTTATTATCTGGTTAT

gi|56599621|gb|CX085631.1|CX08 CTAGTACAAATT-CATTC--TTCGACT-CTCCCAGTTATTATCTGGTTAT

gi|56601669|gb|CX087675.1|CX08 CTAGTACAAATT-CATAC--TTCGACT-CTCCCAGTTATTATCTGGTTAT

gi|56602105|gb|CX088111.1|CX08 CTAGTACAAATT-CATAC--TTCGACT-CTCCCAGTTATTATCTGGTTAT

gi|56602188|gb|CX088194.1|CX08 TTAGTACAAATT-CATTC--TTCGACT-CTCCCAGTTATTATCTGGTTAT

gi|56602405|gb|CX088411.1|CX08 CTAGTACAAATT-CAAAC--TTCGACT-CTCCCAGTTATTATCTGGTTAT

gi|56602598|gb|CX088604.1|CX08 CTAGTACAAATT-CATAC--TTCGACT-CTCCCAGTTATTATCTGGTTAT

gi|56602608|gb|CX088614.1|CX08 CTAGTACAAATT-CATAC--TTCGACT-CTCCCAGTTATTATCTGGTTAT

gi|56603426|gb|CX089432.1|CX08 CTAGTACAAATTTCATTTCTTTCGACTTCTCCCAGTTATTATCTGGTTAT

gi|56615281|gb|CX090913.1|CX09 CTAGTACAAATT-CATTC--TTCGACT-CTCCCAGTTATTATCTGGTTAT

gi|56616332|gb|CX091964.1|CX09 CTAGTACAAATT-CATTC--TTCGACT-CTCCCAGTTATTATCTGGTTAT

gi|56617204|gb|CX092522.1|CX09 TTAGTACAAATT-CAAAC--TTCGACT-CTCACAGTTATTATCTGGTTAT

gi|56617980|gb|CX092894.1|CX09 CTAGTACAAATT-CATAC--TTCGACT-CTCCCAGTTATTATCTGGTTAT

gi|56620877|gb|CX094380.1|CX09 CTAGTACAAATT-CATTC--TTCGACT-CTCCCAGTTATTATCTGGTTAT

gi|56622641|gb|CX095382.1|CX09 TTAGTACAAATT-CATAC--TTCGACT-CTCCCAGTTATTATCTGGTTAT

gi|56623152|gb|CX095893.1|CX09 CTAGTACAAATT-CATTC--TTCGACT-CTCCCAGTTATTATCTGGTTAT

gi|56625177|gb|CX097763.1|CX09 CTAGTACAAATT-CATTC--TTCGACT-CTCCCAGTTATTATCTGGTTAT

gi|56626119|gb|CX098234.1|CX09 CTAGTACAAATT-CAAAC--TTCGACT-CTCCCAGTTATTATCTGGTTAT

gi|56627180|gb|CX098742.1|CX09 CTAGTACAAATT-CATAC--TTCGACT-CTCCCAGTTATTATCTGGTTAT

gi|56627915|gb|CX099120.1|CX09 CTAGTACAAATT-CATAC--TTCGACT-CTCCCAGTTATTATCTGGTTAT

gi|56628353|gb|CX099338.1|CX09 CTAGTACAAATT-CATTC--TTCGACT-CTCCCAGTTATTATCTGGTTAT

*********** ** ******* *** ****** ***********

Consensus_65_2-rep_EhSINE1s GACGGTGCNTTTGAATTAGGAATGTATTAGGGAATGCTGCAAAGGGTGCA

gi|56594149|gb|CX080159.1|CX08 GACGGTGCTTTTGAATTAGGAATGTATTAGGGAATGCTGCAAAGGGTGCA

gi|56594501|gb|CX080511.1|CX08 GACGGTGCCTTTGAATTATGAATGTTTTATGGAATGCTGCAAAGGGTGCA

gi|56594583|gb|CX080593.1|CX08 GACGGTGCCTTTGAATTAGGAATGTATTAGGGAATGCTGCAAAGGGTGTA

gi|56594699|gb|CX080709.1|CX08 GACGGTGCTTTTGAATTAGGAATGTATTAGGGAATGCTGCAAAGGGTGTA

gi|56595210|gb|CX081220.1|CX08 GACGGTGCTTTTGAATTAGGAATGTATTAGGGAATGGTGCAAAGGGTGCA

gi|56595226|gb|CX081236.1|CX08 GACGGTGCCTTTGAATTAGGAATGTATTAGGGAATGCTGCAAAGGGTGCA

gi|56595284|gb|CX081294.1|CX08 GACGGTGCTTTTGAATTAGGAATGTATTAGGGAATGCTGCAAAGGGTGCA

gi|56595554|gb|CX081564.1|CX08 GACGGTGCCTTTGAATTAGGAATGTATTAGGGAATGCTGCAAAGGGTGCA

gi|56595866|gb|CX081876.1|CX08 GACGGTGCTTTTGAATTAGGAATGTATTAGGGAA-GCTGCAAAGGGTGCA

gi|56596052|gb|CX082062.1|CX08 GACGGTGCCTTTGAATTAGGAATGTATTAGGGAATGCTGCAAAGGGTGCA

gi|56596773|gb|CX082783.1|CX08 GACGGTGCCTTTGAATTAGGAATGTATTAGGGAATGCTGCAAAGGGTGCA

gi|56596919|gb|CX082929.1|CX08 GACGGTGCTTTTGAATTAGGAATGTATTAGGGAATGCTGCAAAGGGTGCA

gi|56596986|gb|CX082996.1|CX08 GACGGTGCCTTTGAATTAGGAATGTATTAGGGAATGCTGCAAAGGGTGCA

gi|56597956|gb|CX083966.1|CX08 GACAGTGTATTTGAATTAGGAATGTATTAGGGAATGCTGCAAAGGGTGCA

gi|56598330|gb|CX084340.1|CX08 GACGGTGCCTTTGAATTAGGAATGTATTAGGGAATGCTGCAAAGGGTGCA

gi|56599621|gb|CX085631.1|CX08 GACGGTGCCTTTGAATTAGGAATGTATTAGGGAATGCTGCAAAGGGTGCA

gi|56601669|gb|CX087675.1|CX08 GACAGTGTTTTTGAATTAGGAATGTATTAGGGAATGCTGCAAAGGGTGCA

gi|56602105|gb|CX088111.1|CX08 GACGGTGCTTTTGAATTAGGAATGTATTAGGGAATGCTGCAAAGGGTGCA

gi|56602188|gb|CX088194.1|CX08 GACGGTGCCTTTGAATTAGGAATGTATTAGGGAATGCTGCAAAGGGTGCA

gi|56602405|gb|CX088411.1|CX08 GACGGTGCTTTTGAATTAGGAATGGATTAGGGAATGCTGCAAAGGGTGCA

gi|56602598|gb|CX088604.1|CX08 GACGGTGCCTTTGAATTAGGAATGTATTAGGGAATGCTGCAAAGGGTGCA

gi|56602608|gb|CX088614.1|CX08 GACGGTGCTTTTGAATTAGGAATGTATTAGGGAATGCTGCAAAGGGTGCA

gi|56603426|gb|CX089432.1|CX08 GACGGTGCCTTTGAATTAGGAATGTATTAGGGAATGCTGCAAAGTGTGCA

gi|56615281|gb|CX090913.1|CX09 GACGGTCCTTTTGAATTAGGAATGTATTAGGGAATGCTGCAAAGGGTGCA

gi|56616332|gb|CX091964.1|CX09 GACGGTGCCTTTGAATTAGGAATGTATTAGGGAA-GCTGCAAAGGGTGCA

gi|56617204|gb|CX092522.1|CX09 GACAGTGGCTTTGAATTATGAATGTTTTATGGAATGCTGCAAAGGGTGCA

gi|56617980|gb|CX092894.1|CX09 GACGGTGCCTTTGAATTAGGAATGTATTAGGGAATGCTGCAAAGGGTGCA

gi|56620877|gb|CX094380.1|CX09 GACGGTGCCTTTGAATTAGAAATGTATTAGGGAATGCTGCAAAGGGTGCA

gi|56622641|gb|CX095382.1|CX09 GACGGTGCCTTTGAATTAGGAATGTATTAGGGAATGCTGCAAAGGGTGCA

gi|56623152|gb|CX095893.1|CX09 GACGGTGCCTTTGAATTAGGAATGTATTAGGGAATGCTGCAAAGGGTGCA

gi|56625177|gb|CX097763.1|CX09 GATGGTGCTTTTGAATTAGGAATGTATTAGGGAATGCTGCAAAGGGTGCA

gi|56626119|gb|CX098234.1|CX09 GACGGTGCTTTTGAATTAGGAATGTATTAGGGAATGGTGCAAAGGGTGCA

gi|56627180|gb|CX098742.1|CX09 GACGGTGCCTTTGAATTAGGAATGTATTAGGGAATGCTGCAAAGGGTGCA

gi|56627915|gb|CX099120.1|CX09 GACGGTGCTTTTGAATTAGGAATGTATTAGGGAATGCTGCAAAGGGTGCA

gi|56628353|gb|CX099338.1|CX09 GACGGTGCTTTTGAATTAGGAATGTATTAGGGAATGCTGCAAAGGGTGCA

** ** ********* **** *** **** * ******* *** *

Consensus_65_2-rep_EhSINE1s GCAAGAGAATACAGTAGAATATTACATGGATGTAATATAAGAATCTACTG

gi|56594149|gb|CX080159.1|CX08 GCAAGAGAATACAGTAGAATATTACGTGGATGTAATATAAGAATCTACTG

gi|56594501|gb|CX080511.1|CX08 GCAAGAGAATACAGTAGAATATTACATGGATGTAATATAAGAATATACTG

gi|56594583|gb|CX080593.1|CX08 GCAAGAGAATACAGTAGAATATTACATGGATGTAATATAAGAATATACTG

gi|56594699|gb|CX080709.1|CX08 GCAAGAGAATACAGTAGAATATTACATGGATGTAATATAAGAATCTACTG

gi|56595210|gb|CX081220.1|CX08 GCAAGAGAATACAGTAGAATATTACATGGATGTAATATAAGAATCTACTG

gi|56595226|gb|CX081236.1|CX08 GCAAGAGAATACAGTAGAATATTACGTGGATGTAATATAAGAATCTACTG

gi|56595284|gb|CX081294.1|CX08 GCAAGAGAATACAGTAGAATATTACATGGATGTAATATAAGAATCTACTG

gi|56595554|gb|CX081564.1|CX08 GCAAGAGAATACAGTAGAATATTACATGGATGTAATATAAGAATCTACTG

gi|56595866|gb|CX081876.1|CX08 GCAAGAGAATACAGTAGAATATTACATGGATGTAATATAAGAATCTACTG

gi|56596052|gb|CX082062.1|CX08 GCAAGAGAATACAGTAGAATATTACATG-ATGCAATATAAGAACCTACTG

gi|56596773|gb|CX082783.1|CX08 GCAAGAGAATACAGTAGAATATTACGTGGATGTAATATAAGAATATACTG

gi|56596919|gb|CX082929.1|CX08 GCAAGAGAATACAGTAGAATATTATATGGATGTAATATAAGAATCTACTG

gi|56596986|gb|CX082996.1|CX08 GCAAGAGAATACAGTAGAATATTACATGGATGTAATATAAGAATCTACTG

gi|56597956|gb|CX083966.1|CX08 GCAAGAGAATACAGTAGAATATTACATGGATGTAATATAAGAATCTACTG

gi|56598330|gb|CX084340.1|CX08 GCAAGAGAATACAGTAGAATATTACGTGGATGTAATATAAGAATCTACTG

gi|56599621|gb|CX085631.1|CX08 GCAAGAGAATACAGTAGAATATTACGTGGATGTAATATAAGAATCTACTG

gi|56601669|gb|CX087675.1|CX08 GCAAGATATTATGATAGAATATTACATGGATGTAATATAAGAATCTACTG

gi|56602105|gb|CX088111.1|CX08 GCAAGAGAATACAGTAGAATATTACATGGATGTAATATAAGAATCTACTG

gi|56602188|gb|CX088194.1|CX08 GCAAGAGAATACAGTAGAATATTACATGGATGTAATATAAGAATCTACTG

gi|56602405|gb|CX088411.1|CX08 GCAAGAGAATACAGTAGAATATTACGTGGATGTAATATAAGAATCGACTG

gi|56602598|gb|CX088604.1|CX08 GCAAGAGAATACAGTAGAATATTACATGGATGTAATATAAGAATCTACTG

gi|56602608|gb|CX088614.1|CX08 GCAAGAGAATACAGTAGAATATTACATGGATGTAATATAAGAATCTACTG

gi|56603426|gb|CX089432.1|CX08 GCAAGAGAATACAGTAGAATATTACATGGATGTAATATAAGAATCTACTG

gi|56615281|gb|CX090913.1|CX09 GCAAGAGAATACAGTAGAATATTACATGGATGTAATATAAGAATCTACTG

gi|56616332|gb|CX091964.1|CX09 GCAAGAGAATACAGTAGAATATTACATGGATGTAATATAAGAATCTACTG

gi|56617204|gb|CX092522.1|CX09 GCAAGAGAATACAGTAGAATATTACGTGGATGTAATATAAGAATATACTG

gi|56617980|gb|CX092894.1|CX09 GCAAGAGAATACAGTAGAATATTACATG-ATGCAATATAAGAATCTACTG

gi|56620877|gb|CX094380.1|CX09 GCAAGAGAATACAGTAGAATATTACATGGATGTAATATAAGAATCTACTG

gi|56622641|gb|CX095382.1|CX09 GCAAGAGAATACAGTAGAATATTACATGGATGTAATATAAGAATATACTG

gi|56623152|gb|CX095893.1|CX09 GCAAGAGAATACAGTAGAATATTACATGGATGTAATATAAGAATCTACTG

gi|56625177|gb|CX097763.1|CX09 GCAAGAGAATACAGTAGAATATTACATGGATGTAATATAAGAATCTACTG

gi|56626119|gb|CX098234.1|CX09 GCAAGAGAATACAGTAGAATATTACATGGATGTAATATAAGAATCTACTG

gi|56627180|gb|CX098742.1|CX09 GCAAGAGAATACAGTAGAATATTACGTGGATGTAATATAAGAATCTACTG

gi|56627915|gb|CX099120.1|CX09 GCAAGAGAATACAGTAGAATATTACATGGATGTAATATAAGAACCTACTG

gi|56628353|gb|CX099338.1|CX09 GCAAGAGAATACAGTAGAATATTACATGGATGTAATATAAGAATCTACTG

****** * ** ********** ** *** ********** ****

Consensus_65_2-rep_EhSINE1s AAGTGTGG**GTATGACTAAAAGAAGATTAGTCAAA*GTAAGACTAAAAAGAA***

gi|56594149|gb|CX080159.1|CX08 AAGTGTGGGTATGACTAAAAGAAGATTAGTCAAAGTAAGACTAAAAAGAA

gi|56594501|gb|CX080511.1|CX08 AAGTGTGGGTATGACTAAAAGAAGATTAGTCAAAGTAAGACTAAAAAGAA

gi|56594583|gb|CX080593.1|CX08 AAGTGTGGGTATGACTAAAAGAAGATTAGTCAAAGTAAGACTAAAAAGAA

gi|56594699|gb|CX080709.1|CX08 AAGTGTGGGTATGACTAAAAGAAGATTAGTCAAAGTAAGACTAAAAAGAA

gi|56595210|gb|CX081220.1|CX08 AAGTGTGGGTATGACTAAAAGAAGATTAGTCAAAGTAAGACTAAAAAGAA

gi|56595226|gb|CX081236.1|CX08 AAGTGTGGGTATGACTAAAAGAAGATTAGTCAAAGTAAGACTAAAAAGAA

gi|56595284|gb|CX081294.1|CX08 AAGTGTGGGTATGACTAAAAGAAGATTAGTCAAAGGAAGACTAAAA-GAA

gi|56595554|gb|CX081564.1|CX08 AAGTGTGG---TGACTAAAAGAAGATTAGTCAAAGTAAGACTAAAAAGAA

gi|56595866|gb|CX081876.1|CX08 AGGTGTGGGTATGACTAAAAGAAGATTAGTCAAAGCAAGACTAAAAAGAA

gi|56596052|gb|CX082062.1|CX08 AAGTGTGGGTATGACTAAAAGAAGATTAGTCAAAGTAAGACTAAAAAGAA

gi|56596773|gb|CX082783.1|CX08 AAGTGTGGGTATGACTAAAAGAAGATTAGTCAAAGTAAGACTAAAAAGAA

gi|56596919|gb|CX082929.1|CX08 AAGTGTGGGTATGACTAAAAGAAGATTAGTCAAAGGAAGACTAAAA-GAA

gi|56596986|gb|CX082996.1|CX08 AAGTGTGGGTATGACTAAAAGAAGATTAGTCAAAGTAAGACTAAAAAGAA

gi|56597956|gb|CX083966.1|CX08 AAGTGTGGGTATGACTAAAAGAAGATTAGTCAAAGTAAGACTAAAAAGAA

gi|56598330|gb|CX084340.1|CX08 AAGTGTGGGTATGACTAAGAGAAGATTAGTCAAAGTAAGACTAAAAAGAA

gi|56599621|gb|CX085631.1|CX08 AAGTGTGGGTATGACTAAAAGAAGATTAGTCAAAGTAAGACTAAAAAGAA

gi|56601669|gb|CX087675.1|CX08 AAGTGTGGGTATGACTAAGAGAAGATTAGTCAAAGTAAGACTAAAAAGAA

gi|56602105|gb|CX088111.1|CX08 AAGTGTGGGTATGACTAAAAGAAGATTAGTCAAAGTAAGACTAAAAAGAA

gi|56602188|gb|CX088194.1|CX08 AAGTGTGGGTATGACTAAAAGAAGATTAGTCAAAGTAAGACTAAAAAGAA

gi|56602405|gb|CX088411.1|CX08 AAGTGTGGGTATGACTAAAAGAAGATTAGTCAAAGTAAGACTAAAAAGAA

gi|56602598|gb|CX088604.1|CX08 AAGTGTGGGTATGACTAAAAGAAGATTAGTCAAAGCAAGACTAAAAAGAA

gi|56602608|gb|CX088614.1|CX08 AAGTGTGGGTATGACTAAAAGAAGATTAGTCAAAGTAAGACTAAAAAGAA

gi|56603426|gb|CX089432.1|CX08 AAGTGTGGGTATGACTAAAAGAAGATTAGTCAAAGTAAGACTAAAAAGAA

gi|56615281|gb|CX090913.1|CX09 AAGTGTGGGTATGACTAAAAGAAGATTAGTCAAAGTAAGACTAAAAAGAA

gi|56616332|gb|CX091964.1|CX09 AAGTGTGGGTATGACTAAAAGAAGATTAGTCAAAGTAAGACTAAAAAGAA

gi|56617204|gb|CX092522.1|CX09 AAGTGTGGGTATGACTAAGAGAAGATTAGTCAAAATAAGACTAAAAAGAA

gi|56617980|gb|CX092894.1|CX09 AAGTGTGGGTATGACTAAAAGAAGATTAGTCAAAGTATGACTAAAA-GAA

gi|56620877|gb|CX094380.1|CX09 AAGTGTGGGTATGACTAAAAGAAGATTAGTCAAAGTAAGACTAAAAAGAA

gi|56622641|gb|CX095382.1|CX09 AAGTGTGGGTATGACTAAGAGAAGATTAGTCAAAGTAAGACTAAAAAGAA

gi|56623152|gb|CX095893.1|CX09 AAGTGTGGGTATGACTAAAAGAAGATTAGTCAAAGTAAGACTAAAAAGAA

gi|56625177|gb|CX097763.1|CX09 AAGTGTGGGTATGACTAAAAGAAGATTAGTCAAAGTAAGACTAAAAAGAA

gi|56626119|gb|CX098234.1|CX09 AAGTGTGGGTATGACTAAAAGAAGATTAGTCAAAGTAAGACTAAAAAGAA

gi|56627180|gb|CX098742.1|CX09 AAGTGTGGGTATGACTAAAAGAAGATTAGTCAAAGTAAGACTAAAA-GAA

gi|56627915|gb|CX099120.1|CX09 AAGTGTGAGTATGACTAAAAGAAGATTAGTCAAAGTAAGACTAAAAAGAA

gi|56628353|gb|CX099338.1|CX09 AAGTGTGGGTATGACTAAAAGAAGATTAGTCAAAGTAAGACTAAAAAGAA

* ***** ******* *************** * ******** ***

Consensus_65_2-rep_EhSINE1s ***GATTAGTCAAA***GTAATACAGTAGTAATAAAATGATTCCTTCTNCCATTCA

gi|56594149|gb|CX080159.1|CX08 GATTAGTCAAAGTAATACAGTAGTAATAAAATGATTCCTTCTTCCATTCA

gi|56594501|gb|CX080511.1|CX08 GATTAGTCAAAGTAATACAGTAGTAATAAAATGATTCCTTCTCCCATTCA

gi|56594583|gb|CX080593.1|CX08 AATTAGTCAAAGTAATACAGCAGTAACAAAATGATTCCTTCTCCCATTCA

gi|56594699|gb|CX080709.1|CX08 GATTAGTCAAAGTAATACAGTAGTAATAAAATGATTCCTTCTTCCATTCA

gi|56595210|gb|CX081220.1|CX08 GATTAGTCAAAGTAATACAGTAGTAATAAAATGATTCCTTCTCCCATTCA

gi|56595226|gb|CX081236.1|CX08 GATTAGTCAAAGTAATACAGTAGTAATAAAATGATTCCTTCTTCCATTCA

gi|56595284|gb|CX081294.1|CX08 AATTAGTCAAAGTAATACAGTAGTAATAAAATGATTCCTTCTTCCATTCA

gi|56595554|gb|CX081564.1|CX08 GATTAGTCAAAGTAATATAGTAGTAATAAAATGATTCCTTCTTCCATTCA

gi|56595866|gb|CX081876.1|CX08 AATTAGTCAAAGTAATACAGTAGTAATAAAATAATTCCTTCTCCCATTCA

gi|56596052|gb|CX082062.1|CX08 GATTAGTCAAAGTAATATAGTAGTAATAAAATGATTCCTTCTCCCATTCA

gi|56596773|gb|CX082783.1|CX08 GATTAGTCAAAGTAATACAGTAGTAATAAAATGATTCCTTCTCCCATTCA

gi|56596919|gb|CX082929.1|CX08 AATTAGTCAAAGTAATACAGTAGTAATAAAATGATTCCTTCTTCCATTCA

gi|56596986|gb|CX082996.1|CX08 AATTAGTCAAAGTAATACAGTAGTAATAAAATGATTCCTTCTCCCATTCA

gi|56597956|gb|CX083966.1|CX08 GATTAGTCAAAGTAATACAGTAGTAATAAAATGATTCCTTCTCCCATTCA

gi|56598330|gb|CX084340.1|CX08 GATTAGTCAAAGTAATACAGTAGTAATAAAATGATTCCTTCTCCCATTCA

gi|56599621|gb|CX085631.1|CX08 GATTAGTCAAAGTAATACAGTAGTAATAAAATGATTCCTTCTTCCATTCA

gi|56601669|gb|CX087675.1|CX08 GATTAGTCAAAGTAATACAGTAGTAATAAAATGATTCCTTCTCCTATTCA

gi|56602105|gb|CX088111.1|CX08 AATTAGTCAAAGTAATATAGTAGTAATAAAATGATTCCTTCTTCCATTCA

gi|56602188|gb|CX088194.1|CX08 GATTAGTCAAAGTAATACAGTAGTAATAAAATGATTCCTTCTCCCATTCA

gi|56602405|gb|CX088411.1|CX08 GATTAGTCAAAGTAATATAGTAGTAATGAAACGATTCCTTCTCCCATTCA

gi|56602598|gb|CX088604.1|CX08 GATTAGTCAAAGTAATATAGTAGTAATAAAATGATTCCTTCTTCCATTCA

gi|56602608|gb|CX088614.1|CX08 AATTAGTCAAAGTAATATAGTAGTAATAAAATGATTCCTTCTTCCATTCA

gi|56603426|gb|CX089432.1|CX08 GATTAGTCAAAGTAATACAGTAGTAATAAAATGGTTCCTTCTCCTATTCA

gi|56615281|gb|CX090913.1|CX09 GATTAGTCAAAGTAATACAGTAGTAATAAAATGATTCCTTCTCCCATTCA

gi|56616332|gb|CX091964.1|CX09 GATTAGTCAAAGTAATACAGTAGTAATAAAATGATTCCTTCTTCCATTCA

gi|56617204|gb|CX092522.1|CX09 GATTAGTCAAAGTAATATAGTAGTAATAAAATATTTCCTTCTCCCATTCA

gi|56617980|gb|CX092894.1|CX09 GATTAGTCAAAGTAATACAGTAGTAATGAAATGATTCCTTCTCCCATTCA

gi|56620877|gb|CX094380.1|CX09 GATTAGTCAAAGTAATACAGTAGTAATAAAATGATTCCTTCTTCCATTCA

gi|56622641|gb|CX095382.1|CX09 GATTAGTCAAAGTAATACAGTAGTAATAAAATGATTCCTTCTCCCATTCA

gi|56623152|gb|CX095893.1|CX09 AATTAGTCAAAGTAATACAGTAGTAATAAAATGATTCCTTCTCCCATTCA

gi|56625177|gb|CX097763.1|CX09 GATTAGTCAAAGTAATATAGTAGTAATAAAATGATTCCTTCTCCCATTCA

gi|56626119|gb|CX098234.1|CX09 GATTAGTCAAAGTAATACAGTAGTAATAAAATGATTCCTTCTCCCATTCA

gi|56627180|gb|CX098742.1|CX09 AATTAGTCAAAGTAATATAGTAGTAATAAAATGATTCCTTCTCCCATTCA

gi|56627915|gb|CX099120.1|CX09 GATTAGTCAAAGTAATATAGTAGTAATAAAATGATTCCTTCTCCCATTCA

gi|56628353|gb|CX099338.1|CX09 AATTAGTCAAAGTAATACAGTAGTAATAAAATGATTCCTTCTTCCATTCA

**************** ** ***** *** ******** * *****

Consensus_65_2-rep_EhSINE1s TAAAATAA-GAAAAATGAAATTCCTTAAAATTAAGGCAGAAAACAAACAA

gi|56594149|gb|CX080159.1|CX08 TAAAATAA-GAAAAATGAAATTCTTTAAAATTAAGGCAGAAAACAAACAA

gi|56594501|gb|CX080511.1|CX08 TAAAATAA-GAAAAATGAAATTCCTTAAAATTAAGGCAGAAAACAAACAA

gi|56594583|gb|CX080593.1|CX08 TAAAATAA-GAAAAATGAAATTCCTTAAAATTAAGGTAGAAAACAAACAA

gi|56594699|gb|CX080709.1|CX08 TAAAATAA-GAAAAATGAAATTCCTTAAAATTAAGGCAGAAAACAAACAA

gi|56595210|gb|CX081220.1|CX08 TAAAATAA-GAAAAATGAAATTACTTAAAATTAAGGCAGAAAACAAACAA

gi|56595226|gb|CX081236.1|CX08 TAAAATAA-GAAAAATGAAATTCCTTAAAATTAAGGCAGAAAACAAACAA

gi|56595284|gb|CX081294.1|CX08 TAAAATAA-GAAAAATGAAATTCCTTAAAATTAAGGCAGAAAACAAACAA

gi|56595554|gb|CX081564.1|CX08 TAAAATAA-GAAAAATGAAATTCCTTAAAATTAAGGTAGAAAACAAACAA

gi|56595866|gb|CX081876.1|CX08 TAAAATAA-GAAAAATGAAATTCCTTAAAATTAAGGCAGAAAACAAACAA

gi|56596052|gb|CX082062.1|CX08 TAAAATAA-GAAAAATGAAATTCCTGAAAATTAAGGCAGAAAACAAACAA

gi|56596773|gb|CX082783.1|CX08 TAAAATAA-GAAAAATGAAATTCCTGAAAATTAAGGCAGAAAATAAACAA

gi|56596919|gb|CX082929.1|CX08 TAAAATAA-GAAAAATGAAATTCCTTAAAATTAAGGCAGAAAACAAACAA

gi|56596986|gb|CX082996.1|CX08 TAAAATAA-GAAAAATGAAATTACTTAAAATTAAGGCAGAAAACAAACAA

gi|56597956|gb|CX083966.1|CX08 TAAAATAA-GAAAAATGAAATTCCTTAAAATTAAGGCAGAAAACAAACAA

gi|56598330|gb|CX084340.1|CX08 TAAAATAA-GAAAAATGAAATTCCTTAAAATTAAGGCAGAAAACAAACAA

gi|56599621|gb|CX085631.1|CX08 TAAAATAA-GAAAAATGAAATTCCTTAAAATTAAGGCAGAAAACAAACAA

gi|56601669|gb|CX087675.1|CX08 TAAAATAAAGGAAAAAGAAAATGTTTAAAATTAAGGCAGAAAACAAACAA

gi|56602105|gb|CX088111.1|CX08 TAAAATAA-GAAAAATGAAATTCCTTAAAATTAAGGTAGAAAACAAACAA

gi|56602188|gb|CX088194.1|CX08 TAAAATAA-GAAAAATGAAATTCCTGAAAATTAAGGCAGAAAACAAACAA

gi|56602405|gb|CX088411.1|CX08 TAAAATAA-GAAAAATGAAATTCCTTAAAATTAAGGCAGAAAACAAACAA

gi|56602598|gb|CX088604.1|CX08 TAAAATAA-GAAAAATGAAATTCCTGAAAATTAAGGCAGAAAACAAACAA

gi|56602608|gb|CX088614.1|CX08 TAAAATAA-GAAAAATGAAATTCCTTAAAATTAAGGCAGAAAACAAACAA

gi|56603426|gb|CX089432.1|CX08 TAAAATAA-GAAAAATGAAATTACTTAAAATTAAGGCAGAAAACAAACAA

gi|56615281|gb|CX090913.1|CX09 TAAAATAA-GAAAAATGAAATTCCTTAAAATTAAGGCAGAAAACAAACAA

gi|56616332|gb|CX091964.1|CX09 TAAAATAA-GAAAAATGAAATTCCTTAAAATTAAGGCAGAAAACAAACAA

gi|56617204|gb|CX092522.1|CX09 TAAAATAA-GAAAAATGAAATTCCTTAAAATTAAGGCAGAAAACAAACAA

gi|56617980|gb|CX092894.1|CX09 TAAAATAA-GAAAAATGAAATTCCTTAAAATTAAGGCAGAAAACAAACAA

gi|56620877|gb|CX094380.1|CX09 TAAAATAA-GAAAAATGAAATTCCTTAAAATTAAGGCAGAAAACAAACAA

gi|56622641|gb|CX095382.1|CX09 TAAAATAA-GAAAAATGAAATTCCTGAAAATTAAGGCAGAAAACAAACAA

gi|56623152|gb|CX095893.1|CX09 TAAAATAA-GAAAAATGAAATTCCTTAAAATTAAGGCAGAAAACAAACAA

gi|56625177|gb|CX097763.1|CX09 TAAAATAA-GAAAAATGAAATTCCTTAAAATTAAGGCAGAAAACAAACAA

gi|56626119|gb|CX098234.1|CX09 TAAAATAA-GAAAAATGAAATTACTTAAAATTAAGGCAGAAAACAAACAA

gi|56627180|gb|CX098742.1|CX09 TAAAATAA-GAAAAATGAAATTCCTTAAAATTAAGGCAGAAAACAAACAA

gi|56627915|gb|CX099120.1|CX09 TAAAATAA-GAAAAATGAAATTCCTGAAAATTAAGGCAGAAAATAAACAA

gi|56628353|gb|CX099338.1|CX09 TAAAATAA-GAAAAATGAAATTCCTTAAAATTAAGGCAGAAAACAAACAA

******** * **** **** * * ********** ****** ******

Consensus_65_2-rep_EhSINE1s AGGCTTAAAAA--GAAGAAATAAGCAGAAGAAGTTTGAAAAA-CCTTAAT

gi|56594149|gb|CX080159.1|CX08 AGGCTTAAAAA--GAAGAAATAAGCAGAAGAAGTTTGAAAAAACCTTAAT

gi|56594501|gb|CX080511.1|CX08 AGGCTTAAAAA--GAAGAAATAAGCAGAAGAAGTTTGAAAAA-CCTTAAT

gi|56594583|gb|CX080593.1|CX08 AGGCTTAAAAA--GAAGAAATAAGCAGAAGAAGTTTGAAAAA-CCTTAAT

gi|56594699|gb|CX080709.1|CX08 AGGCTTAAAAA--GAAGAAATAAGCAGAAGAAGTTTGAAAAA-CCTTAAT

gi|56595210|gb|CX081220.1|CX08 AGGCTTAAAAA--GAAGAAATAAGCAGAAGAAGTTTGAAAAAACCTTAAT

gi|56595226|gb|CX081236.1|CX08 AGGCTTAAAAA--GAAGAAATAAGCAGAAGAAGTTTGAAAAA-CCTTAAT

gi|56595284|gb|CX081294.1|CX08 AGGCTTAAAAA--GAAGAATTAAGCAGAAGAAGTTTGAAAAA-CCTTAAT

gi|56595554|gb|CX081564.1|CX08 AGGCTTAAAAA--GAAGAAATAAGCAGAAGAAGTTTGAAAAAACCTTAAT

gi|56595866|gb|CX081876.1|CX08 AGGCTTAAAAA--GAAGAAATAAGCAGAAGAAGTTTGAAAAA-CCTTAAT

gi|56596052|gb|CX082062.1|CX08 AGGCTTAAAAA--GAAGAAATAAGCAGAAGAAGTTTGAAAAA-CCTTAAT

gi|56596773|gb|CX082783.1|CX08 AAGGTTAAAAA--GAAGAAATAAGCAGAAGAAGTTTGAAAAA-CCTTAAT

gi|56596919|gb|CX082929.1|CX08 AGGCTTAAAAA--GAAGAAATAAGCAGAAGAAGTTTGAAAAA-CCTTAAT

gi|56596986|gb|CX082996.1|CX08 AGGCTTAAAAA--GAAGAATTAAGCAGAAGAAGTTTGAAAAA-CCTTAAT

gi|56597956|gb|CX083966.1|CX08 AGGCTTAAAAA--GAAGAAATAAGCAGAAGAGGTTTGAAAAA-CCTTAAT

gi|56598330|gb|CX084340.1|CX08 AGGCTTAAAAA--GAAGAATTAAGCAGAAGAAGTTTGAAAAA-CCTTAAT

gi|56599621|gb|CX085631.1|CX08 AGGCTTAAAAA--GAAGAAATAAGCAGAAGAAGTTTGAAAAA-CCTTAAT

gi|56601669|gb|CX087675.1|CX08 AATGTTAAAAA--GAAGAAATAAGCAGAA-----TTGAAAGA-CATTAAT

gi|56602105|gb|CX088111.1|CX08 AGGCTTAAAAA--GAAGAAATAAGCAGAAGAAGTTTGAAAAAACCTTAAT

gi|56602188|gb|CX088194.1|CX08 AGGCTTAAAAA--GAAGAAATAAGCAGAAGAAGTTTGAAAAAACCTTAAT

gi|56602405|gb|CX088411.1|CX08 AGGCTTAAAAA--GAAGAAATAAGCAGAAGAAGTTTGAAAAA-CCTTAAT

gi|56602598|gb|CX088604.1|CX08 AGGCTTAAAAA--GAAGAAATAAGCAGAAGAAGTTTGAAAAAACCTTAAT

gi|56602608|gb|CX088614.1|CX08 AGGCTTAAAAA--GAAGAATTAAGCAGAAGAAGTTTGAAAAA-CCTTAAT

gi|56603426|gb|CX089432.1|CX08 AAGCTTAAAAA--GAAGAAATAAGCAGAAGAAGTTTGAAAAA-CCTTAAT

gi|56615281|gb|CX090913.1|CX09 AGGCTTAAAAA--GAAGAAATAAGCAGAAGAGGTTTGAAAAA-CCTTAAT

gi|56616332|gb|CX091964.1|CX09 AGGCTTAAAAA--GAAGAAATAAGCAGAAGAAGTTTGAAAAAACCTTAAT

gi|56617204|gb|CX092522.1|CX09 AGGCTTAAAAA--GAAGAAATAAGCAGAAGAAGTTTGAAAAA-CCTTAAT

gi|56617980|gb|CX092894.1|CX09 AGGCTTAAAAA--GAAGAAATAAGCAGAAGAAGTTTGAAAAA-CCTTAAT

gi|56620877|gb|CX094380.1|CX09 AGGCTTAAAAA--GAAGAAATAAGCAGAAGAAGTTTGAAAAA-CCTTAAT

gi|56622641|gb|CX095382.1|CX09 AGGCTTAAAAA--GAAGAAATAAGCAGAAGAAGTTTGAAAAA-CCTTAAT

gi|56623152|gb|CX095893.1|CX09 AGGTTTAAAAA--GAAGAAATAAGCAGAAGAAGTTTGAAAAA-CCTTAAT

gi|56625177|gb|CX097763.1|CX09 AGGCTTAAAAA--GAAGAAATAAGCAGAAGAAGTTTGAAAAA-CTTTAAT

gi|56626119|gb|CX098234.1|CX09 AGGCTTAAAAA--GAAGAAATAAGCAGAAGAAGTTTGAAAAAACCTTAAT

gi|56627180|gb|CX098742.1|CX09 AGGCTTAAAAAAAGAAGAAATAAGCAGAAGAAGTTTGAAAAAACCTTAAT

gi|56627915|gb|CX099120.1|CX09 AGGCTTAAAAA--GAAGAAATAAGCAGAAGAAGTTTGAAAAA-CCTTAAT

gi|56628353|gb|CX099338.1|CX09 AGGCTTAAAAA--GAAGAATTAAGCAGAAGAAGTTTGAAAAAACCTTAAT

* ******* ****** ********* ****** * * *****

Consensus_65_2-rep_EhSINE1s AGGAAGAAATAAAGCAAAGAAGTGCTTTCCTCATTTTGCAAGANAAACNT

gi|56594149|gb|CX080159.1|CX08 AGGAA-AAATAAAGCAAAGAAGTGCTTTCCTCATTTTGCAAGAAAAACAT

gi|56594501|gb|CX080511.1|CX08 AGGAAGAAATAAAGGAAAGAAGTGCTTTCCTCATTTTGCAAGAAAAACAT

gi|56594583|gb|CX080593.1|CX08 AAGAAGAAATAAAGCAAAGAAGTGCTTTCCTCATTTTGCAAGAAAAACAT

gi|56594699|gb|CX080709.1|CX08 AGGAAGAAATAAAGGAAAGAAGTGCTTTCCTCATTTTGCAAGAAAAACAT

gi|56595210|gb|CX081220.1|CX08 AGGAAGAAATAAAGCAAAGAAGTGGTTTCCTCATTTTGCAAGAAAAACAT

gi|56595226|gb|CX081236.1|CX08 AGGAA-AAATAAAGCAAAGAAGTGCTTTCCTCATTTTGCAAGACAAACCT

gi|56595284|gb|CX081294.1|CX08 AGGAA-AAATAAAGCAAAGAAGTGCTTTCCCCATTTTGCAAGACAAACCT

gi|56595554|gb|CX081564.1|CX08 AGGAA-AAATAAAGCAAAGAAGTGGTTTCCTCATTTTGCAAGAAAAACCT

gi|56595866|gb|CX081876.1|CX08 AAGAAGAAATAAAAGAAAGAAGTGCTTTCCTCATTTTGCAAAACAAACCT

gi|56596052|gb|CX082062.1|CX08 AGGAAGAAATAAAGCAAAGAAGTGCTTTCCTCAATTTGCAAGAAAAACAT

gi|56596773|gb|CX082783.1|CX08 AAGAAGAAATAAAACAAAGAAGTGCTTTCCTCATTTTGCAAGAAAAACAT

gi|56596919|gb|CX082929.1|CX08 AGGAA-AAATAAAACAAAGAAGTGCTTTCCTCATTTTGCAAGAAAAACCT

gi|56596986|gb|CX082996.1|CX08 AAGAAGAAATAAAACAAAGAAGTGCTTTCCTCATTTTGCAAGAAAAACAT

gi|56597956|gb|CX083966.1|CX08 AGGAAGAAATAAAGCAAAGAAGTGCTTTCCTCATTTTGCAAGAAAAATCT

gi|56598330|gb|CX084340.1|CX08 AGGAAGAAATAAAGGAAAGAAGTGCTTTCCTCAATTTCCAATACAAACCT

gi|56599621|gb|CX085631.1|CX08 AGGAA-AAATAAAGCAAAGAAGTGCTTTCCTCATTTTGCAAGACAAACCT

gi|56601669|gb|CX087675.1|CX08 AAGAA-AAATAAAGCAAATAAATGATTTTCTCATTTTCCAAGACAAACCT

gi|56602105|gb|CX088111.1|CX08 AGGAA-AAATAAAGCAAAGAAGTGCTTTCCTCATTTTGCAAGACAAACCT

gi|56602188|gb|CX088194.1|CX08 AGGAAGAAATAAAGCAAAGAAGTGGTTTCCTCATTTTGCAAGAAAAACAT

gi|56602405|gb|CX088411.1|CX08 AGGAAGAAATAAAAGAAAGAAGTGCTTTCCTCAAATTCCAAGACAAACCC

gi|56602598|gb|CX088604.1|CX08 AGGAA-AAATAAAGCAAAGAAGTGGTTTCCTCATTTTGCAAGAAAAACAT

gi|56602608|gb|CX088614.1|CX08 AGGAA-AAATAAAGCAAATAAGTGGTTTCCTCATTTTGCAAGACAAACCT

gi|56603426|gb|CX089432.1|CX08 AGGAA-AAATAAAGCAAAGAAGTGGTTTCCTCATTTTGCAAGAAAAACAT

gi|56615281|gb|CX090913.1|CX09 AGGAAGAAATAAAGCAAAGAAGTGCTTTCCTCATTTTGCAAGAAAAACAT

gi|56616332|gb|CX091964.1|CX09 AGGAA-AAATAAAGCAAAGAAGTGCTTTCCTCATTTTGCAAGACAAACCT

gi|56617204|gb|CX092522.1|CX09 AGGAAGAAATAAAGGAAAGAAGTGCTTTCCTCATTTTGCAAGAAAAACAT

gi|56617980|gb|CX092894.1|CX09 AGGAAGAAATAAAGCAAAGAAGTGCTTTCCTCAATTTGCAAGAAAAACAT

gi|56620877|gb|CX094380.1|CX09 AGGAA-AAATAAAACAAAGAAGTGCTTTCCTCATTTTGCAAGACAAACCT

gi|56622641|gb|CX095382.1|CX09 AGGAAGAAATAAAACAAAGAAGTGCTTTCCTCATTTTGCAAGAAAAACAT

gi|56623152|gb|CX095893.1|CX09 AGGAAGAAATAAAGGAAAGAAGTGCTTTCCTCATTTTGCAAGAAAAACAT

gi|56625177|gb|CX097763.1|CX09 AAGAAGAAATAAAAGAAAGAAGTGCTTTCCTCATTTTGCAAGACAAACCT

gi|56626119|gb|CX098234.1|CX09 AGGAAGAAATAAAGCAAAGAAGTGGTTTCCTCATTTTGCAAGAAAAACAT

gi|56627180|gb|CX098742.1|CX09 AGGAA-AAATAAAACAAAGAAATACTTTCCTCATTTTGCAAGACAAACCT

gi|56627915|gb|CX099120.1|CX09 AAGAAGAAATAAAGCAAAGAAGTGGTTTCCTCATTTTGCAAGAAAAACAT

gi|56628353|gb|CX099338.1|CX09 AGGAA-AAATAAAGCAAAGAAGTGCTTTCCTCATTTTGCAAGACAAACCT

* *** ******* *** ** * *** * ** ** *** * ***

Consensus_65_2-rep_EhSINE1s AAANNATAGGTTNAACAAAGAGATTACTCTTTTTTAATAA---GCTCAGG

gi|56594149|gb|CX080159.1|CX08 AAATGATAGGTTTAACAAAGAGATTACTCTTTTTTAATAA---GCTCAGG

gi|56594501|gb|CX080511.1|CX08 AAAGAATAGGTTTAACAAAGAGATTACTCTTTTTTAATAA---GCTCAGG

gi|56594583|gb|CX080593.1|CX08 AAAGAATAGGTTTAACAAAGAGATTACTCTTTTTTAATAATAAGCTCAGG

gi|56594699|gb|CX080709.1|CX08 AAAGAATAGGTTTAACAAAGAGATTACTCTTTTTTAATAA---GCTCAGG

gi|56595210|gb|CX081220.1|CX08 AAAGAATAGGTTTAACAAAGAGATTACTCTTTTTTAATAA---GCTCAGG

gi|56595226|gb|CX081236.1|CX08 AAATGATAGGTTCAACAAAGAGATTACTCTTTTTTAATAA---GCTCAGG

gi|56595284|gb|CX081294.1|CX08 AAATGATAGGTTAAACAAAGAGATTACTCTTTTTTAAT---AAGCTCAGG

gi|56595554|gb|CX081564.1|CX08 AAATGATAGGTTAAACAAAGAGATTACTCTTTTTTAATAATAAGCTCAGG

gi|56595866|gb|CX081876.1|CX08 AAATGATAGGTTCAACAAAGAGATTACTCTTTTTTAATAA---GCTCAGG

gi|56596052|gb|CX082062.1|CX08 AAATGATAAGTTAAAGAAAAAGATTACTCTTTTTTAATAA---GCTCAGG

gi|56596773|gb|CX082783.1|CX08 AAAGAATAGGTTTAACAAAGAGATTACTCTTTTTTAATAATAAGCTCAGG

gi|56596919|gb|CX082929.1|CX08 AAATGATAGGTTAAACAAAGAGATTACTCTTTTTTAAT---AAGCTCAGG

gi|56596986|gb|CX082996.1|CX08 AAAGAATAGGTTTAACAAAGAGATTACTCTTTTTTAATAA---GCTCAGG

gi|56597956|gb|CX083966.1|CX08 AAATGATAAGTTAAACAAAGAGATTACTCTTTTTTAATAA---GCTCAGG

gi|56598330|gb|CX084340.1|CX08 AAATGATAGGTTCAACAAAGAGATTACTCTTTTTTAATAA---GCTCAGG

gi|56599621|gb|CX085631.1|CX08 AAATGATAGGTTCAACAAAGAGATTACTCTTTTTTAATAA---GCTCAGG

gi|56601669|gb|CX087675.1|CX08 AAATGATAGGTTAAACAAAGAGATTACTCTTTTTTAATAA---GCGAAGG

gi|56602105|gb|CX088111.1|CX08 AAATGATAGGTTAAACAAAGAGATTACTCTTTTTTAATAATAAGCTCAGG

gi|56602188|gb|CX088194.1|CX08 AAAGAATAGGTTTAACAAAGAGATTACTCTTTTTTAATAA---GCTCAGG

gi|56602405|gb|CX088411.1|CX08 AAAGAATAGGTTAAACAAAGAGATTACTCTTTTTTAATAATAGGCGAAGG

gi|56602598|gb|CX088604.1|CX08 AAAGAATAGGTTTAACAAAGAGATTACTCTTTTTTAATAATAAGCTCAGG

gi|56602608|gb|CX088614.1|CX08 AAATGATAGGTTAAACAAAGAGGTTACTCTTTTTTAAT---AAGCTCAGG

gi|56603426|gb|CX089432.1|CX08 AAATGATAGGTTTAACAAAGAGATTACTCTTTTTTAATAA---GCTCAGG

gi|56615281|gb|CX090913.1|CX09 AAAGAATAGGTTTAACAAAGAGATTACTCTTTTTTAATAA---GCTCAGG

gi|56616332|gb|CX091964.1|CX09 AAATGATAGGTTCAACAAAGAGATTACTCTTTTTTAATAA---GCTCAGG

gi|56617204|gb|CX092522.1|CX09 AAAGAATAGGTTTAACAAAGAGATTACTCTTTTTTAATAA---GCTCAGG

gi|56617980|gb|CX092894.1|CX09 AAAGAATAGGTTTAACAAAGAGATTACTCTTTTTTAATAA---GCTCAGG

gi|56620877|gb|CX094380.1|CX09 AAATGATAGGTTAAACAAAGAGATTACTCTTTTTTAAT---AAGCTCAGG

gi|56622641|gb|CX095382.1|CX09 AAAGAATAGGTTTAACAAAGAGATTACTCTTTTTTAATAA---GCTCAGG

gi|56623152|gb|CX095893.1|CX09 AAAGAATAGGTTTAACAAAGAGATTACTCTTTTTTAATAA---GCTCAGG

gi|56625177|gb|CX097763.1|CX09 AAATGATAGGTTCAACAAAGAGATTACTCTTTTTTAAT---AAGCTCAGG

gi|56626119|gb|CX098234.1|CX09 AAAGAATAGGTTTAACAAAGAGATTACTCTTTTTTAATAA---GCTCAGG

gi|56627180|gb|CX098742.1|CX09 AAATGATAGGTTAAACAAAGAGATTACTCTTTTTTAATAA---GCTCAGG

gi|56627915|gb|CX099120.1|CX09 AAAGAATAGGTTTAACAAAGAGATTACTCTTTTTTAATAA---GCTCAGG

gi|56628353|gb|CX099338.1|CX09 AAATGATAGGTTAAACAAAGAGATTACTCTTTTTTAAT---AAGCTCAGG

*** *** *** ** *** ** *************** ** ***

Consensus_65_2-rep_EhSINE1s GATGGGATTAGTCTCCCCTGAGCTAGGAAGAATAGATGAAAATTCTATTA

gi|56594149|gb|CX080159.1|CX08 GATGGGATTAGTCTCCCCTGAGCTAGGAAGAATAGATGAAAATTCTATTA

gi|56594501|gb|CX080511.1|CX08 GATGGGATTAGTCTCCCCTGAGCTAGGAAGAATAGATGAAAATTCTATTA

gi|56594583|gb|CX080593.1|CX08 GATGGGATTAGTCTCCCCTGAGCTAGGAAGAATAGATGAAAATTCTATTA

gi|56594699|gb|CX080709.1|CX08 GATGGGATTAGTCTCCCCTGAGCTAGGAAGAATAGATGAAAATTCTATTA

gi|56595210|gb|CX081220.1|CX08 GATGGGATTAGTCTCCCCTGAGCTAGGAAGAATAGATGAAAAATCTATTA

gi|56595226|gb|CX081236.1|CX08 GATGGGATTAGTCTCCCCTGAGCTAGGAAGAATAGATGAAAATTCCATTA

gi|56595284|gb|CX081294.1|CX08 GATGGGATTAGTCTCCCCTGAGCTAGGAAGAATAGATGAAAATTCTATTA

gi|56595554|gb|CX081564.1|CX08 GATGGGATTAGTCTCCCCTGAGCTAGGAAGAATAGATGAAAATTCTATTA

gi|56595866|gb|CX081876.1|CX08 GATGGGATTAGTCTCCCCTGAGCTAGGAAGAATAGATGAAAAATCTATTA

gi|56596052|gb|CX082062.1|CX08 GATGGGATTAGTCTCCCCTGAGCTAGGAAGAATAGATGAAAAATCTATTA

gi|56596773|gb|CX082783.1|CX08 GATGGGATTAGTCTCCCCTGAGCTAGGAAGAATAGATGAAAATTCTATTA

gi|56596919|gb|CX082929.1|CX08 GATGGGATTAGTCTCCCCTGAGCTAGGAAGAATAGATGAAAATTCTATTA

gi|56596986|gb|CX082996.1|CX08 GATGGGATTAGTCTCCCCTGAGCTAGGAAGAATAGATGAAAATTCTATTA

gi|56597956|gb|CX083966.1|CX08 GATGGGATTAGTCTCCCCTGAGCTAGGAAGAATAGATGAAAATTCTATTA

gi|56598330|gb|CX084340.1|CX08 GATGGGATTAGTCTCCCCTGAGCTAGGAAGAATAGATGAAAATTCTATTA

gi|56599621|gb|CX085631.1|CX08 GATGGGATTAGTCTCCCCTGAGCTAGGAAGAATAGATGAAAATTCCATTA

gi|56601669|gb|CX087675.1|CX08 GATGGGATTAATCTCCTCTAAACTAGGAAGAATAGATGAAAAATCTATTA

gi|56602105|gb|CX088111.1|CX08 GATGGGATTAGTCTCCCCTGAGCTAGGAAGAATAGATGAAAATTCTATTA

gi|56602188|gb|CX088194.1|CX08 GATGGGATTAGTCTCCCCTGAGCTAGGAAGAATAGATGAAAATTCTATTA

gi|56602405|gb|CX088411.1|CX08 GATGGGATTAGTCTCCCCTGAGCTAGGAAGAATAGATGAAAATTCTATTA

gi|56602598|gb|CX088604.1|CX08 GATGGGATTAGTCTCCCCTGAGCTAGGAAAAATAGATGAAAAATCTATTA

gi|56602608|gb|CX088614.1|CX08 GATGGGATTAGTCTCCCCTGAGCTAGGAAGAATAGATGAAAATTCTATTA

gi|56603426|gb|CX089432.1|CX08 GATGGGATTAGTCTCCCCTGAGCTAGGAAGAATAGATGAAAATTCTATTA

gi|56615281|gb|CX090913.1|CX09 GATGGGATTAGTCTCCCCTGAGCTAGGAAGAATAGATGAAAATTCTATTA

gi|56616332|gb|CX091964.1|CX09 GATGGGATTAGTCTCCCCTGAGCTAGGAAGAATAGATGAAAATTCTATTA

gi|56617204|gb|CX092522.1|CX09 GATGGGATTAGTCTCCCCTGAGCTAGGAAGAATAGATGAAAATTCTATTA

gi|56617980|gb|CX092894.1|CX09 GATGGGATTAGTCTCTCCTGAGCTAGGAAGAATATATGAAAATTCTATTA

gi|56620877|gb|CX094380.1|CX09 GATGGGATTAGTCTCCTCTGAGCTAGGAAGAATAGATGAAAATTCTATTA

gi|56622641|gb|CX095382.1|CX09 GATGGGATTAGTCTCCCCTGAGCTAGGAAGAATAGATGAAAAATCTATTA

gi|56623152|gb|CX095893.1|CX09 GATGGGATTAGTCTCCCCTGAGCTAGGAAGAATAGATGAAAATTCTATTA

gi|56625177|gb|CX097763.1|CX09 GATGGGATTAGTCTCCCCTGAGCTAGGAAGAATAGATGAAAATTCTATTA

gi|56626119|gb|CX098234.1|CX09 GATGGGATTAGTCTCCCCTGAGCTAGGAAGAATAGATGAAAAATCTATTA

gi|56627180|gb|CX098742.1|CX09 GATGGGATTAGTCTCCCCTGAGCTAGGAAGAATAGATGAAAATTCTATTA

gi|56627915|gb|CX099120.1|CX09 GATGGGATTAGTCTCCCCTGAGCTAGGAAGAATAGATGAAAAATCTATTA

gi|56628353|gb|CX099338.1|CX09 GATGGGATTAATCTCCCCTGAGCTAGGAAGAATAGATGAAAATTCTATTA

********** **** ** * ******* **** ******* ** ****

Consensus_65_2-rep_EhSINE1s ATACTTAATTAANTANTTTT--------------

gi|56594149|gb|CX080159.1|CX08 ATACTTAATTAACTACTTTTTATTTT--------

gi|56594501|gb|CX080511.1|CX08 ATACTTAATTAACTAATTTTTCTTCTTAAAA---

gi|56594583|gb|CX080593.1|CX08 ATACTTAATTAATTACTTTTTCTTT---------

gi|56594699|gb|CX080709.1|CX08 ATACTTAATTAATTACTTTTTCTTTTTAAAA---

gi|56595210|gb|CX081220.1|CX08 ATACTTAATTAATTACTTTTTCTTTCT-------

gi|56595226|gb|CX081236.1|CX08 ATACTTAATTAACTAATTTTTATTT---------

gi|56595284|gb|CX081294.1|CX08 ATACTTAATTAACTAATTTTTATT----------

gi|56595554|gb|CX081564.1|CX08 ATACTTAATTAACTAATTTTTATTTT--------

gi|56595866|gb|CX081876.1|CX08 ATACTTAATTAATTACTTTTTCTTTT--------

gi|56596052|gb|CX082062.1|CX08 ATACTTAATTAATTACTTTTTATTT---------

gi|56596773|gb|CX082783.1|CX08 ATACTTAATTAATTACTTTTTATTTTT-------

gi|56596919|gb|CX082929.1|CX08 ATACTTAATTAACTAATTTTTATTT---------

gi|56596986|gb|CX082996.1|CX08 ATACTTAATTAATTACTTTTTCTTC---------

gi|56597956|gb|CX083966.1|CX08 ATACTTAATTAACTAATTTTTATTT---------

gi|56598330|gb|CX084340.1|CX08 ATACTTAATTAACTAATTTTTATTAAAA------

gi|56599621|gb|CX085631.1|CX08 ATACTTAATTAACTAATTTTTATTT---------

gi|56601669|gb|CX087675.1|CX08 ATACTTAATTAATTACTTTTTATTC---------

gi|56602105|gb|CX088111.1|CX08 ATACTTAATTAACTAATTTTTATTT---------

gi|56602188|gb|CX088194.1|CX08 ATACTTAATTAACTAATTTTTATTT---------

gi|56602405|gb|CX088411.1|CX08 ATACTTAATTAATTACTTTTTCTTTC--------

gi|56602598|gb|CX088604.1|CX08 ATACTTAATTAATTACTTTTTATTT---------

gi|56602608|gb|CX088614.1|CX08 ATACTTAATTAACTAATTTTTATT----------

gi|56603426|gb|CX089432.1|CX08 ATACTTAATTAACTAATTTTTATTTTAA------

gi|56615281|gb|CX090913.1|CX09 ATACTTAATTAACTAATTTTTATTT---------

gi|56616332|gb|CX091964.1|CX09 ATACTTAATTAACTAATTTTTATTT---------

gi|56617204|gb|CX092522.1|CX09 ATACTTAATTAATTACTTTTTATTTA--------

gi|56617980|gb|CX092894.1|CX09 ATACTTAATTAATTACTTTTTATTTAAAA-----

gi|56620877|gb|CX094380.1|CX09 ATACTTAATTAACTAATTTTTATTT---------

gi|56622641|gb|CX095382.1|CX09 ATACTTAATTAACTAATTTTTATTTT--------

gi|56623152|gb|CX095893.1|CX09 ATACTTAATTAACTAATTTTTATTT---------

gi|56625177|gb|CX097763.1|CX09 ATACTTAATTAACTAATTTATATATTTTTTATGA

gi|56626119|gb|CX098234.1|CX09 ATACTTAATTAATTACTTTTTCTTT---------

gi|56627180|gb|CX098742.1|CX09 ATACTTAATTAATTGATTATTATT----------

gi|56627915|gb|CX099120.1|CX09 ATACTTAATTAATTACTTTTTCTT----------

gi|56628353|gb|CX099338.1|CX09 ATACTTAATTAACTAATTTTTATGA---------

************ * **
